# CA3 Pyramidal Neuron Activation Promotes Cognitive Resilience to Inflammation‐Induced Cognitive Inflexibility

**DOI:** 10.1111/cns.70271

**Published:** 2025-02-25

**Authors:** Wenqiang Zuo, Suwen Fang, Tiantian Xu, Yumeng Li, Jianshuai Zhao, Xiaoyan Xie, Taozhi Wang, Wugang Hou, Minghui Wang

**Affiliations:** ^1^ Department of Anesthesiology and Perioperative Medicine, Xijing Hospital The Fourth Military Medical University Xi'an China

**Keywords:** cognitive flexibility, cognitive resilience, cornu ammonis area 3, IL‐1β, systemic inflammation

## Abstract

**Aims:**

Cognitive dysfunction represents a prominent symptom in numerous prevalent mental illnesses, with systemic inflammation induced by cytokines recognized as a critical factor contributing to cognitive impairments. However, a significant proportion of individuals exposed to systemic inflammation do not develop cognitive dysfunction; instead, they exhibit adaptive responses to this adverse condition. This study aims to investigate the neural activity patterns within the hippocampus and the potential mechanisms that mediate cognitive resilience, particularly in the context of inflammation.

**Methods:**

We investigated the effects of systemic IL‐1β (Interleukin‐1β) on learning, spatial memory, and cognitive flexibility using the Barnes maze test (BMT). We further analyzed specific activity changes in the hippocampus of mice exhibiting cognitive resilience versus susceptibility through immunofluorescence, fiber photometry, and behavioral assessments. Additionally, we employed chemogenetic modulation to explore the role of dCA3 pyramidal neurons in cognitive inflexibility induced by systemic inflammation.

**Results:**

Systemic inflammation induces cognitive inflexibility while leaving learning and memory intact. Notably, dCA3 activity was elevated in cognitively resilient mice compared to their susceptible counterparts. Fiber photometry data revealed higher activity in the dorsal CA3 (dCA3) when the mice approached the previous target quadrant during the reversal stage of BMT. Importantly, the activation of CaMKII^+^ pyramidal neurons in the dCA3 mitigated cognitive inflexibility induced by systemic IL‐1β administration.

**Conclusions:**

Activation of hippocampal dCA3 neurons, rather than dentate gyrus (DG) neurons, enhances cognitive resilience by improving cognitive flexibility during BMT‐related paradigm shifting under sustained inflammation.

## Graphical abstract

Prolonged exposure to IL‐1β impairs cognitive flexibility in animals, evidenced by poor performance in the Barnes maze reversal phase. Mice with cognitive resilience show increased neuronal excitability in the dCA3. Activation of CA3 pyramidal neurons improves cognitive flexibility, while inhibition reproduces the cognitive inflexibility linked to IL‐1β administration.

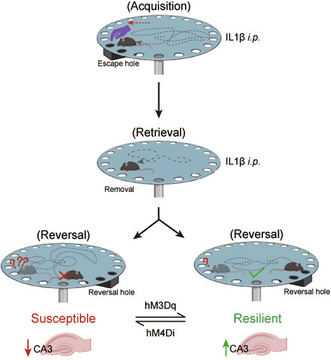

## 1 Introduction

Cognitive resilience refers to an individual's ability to adapt to the negative effects of setbacks and the associated environmental or intrinsic stressors on cognitive function [1]. Prolonged reductions in cognitive resilience have been linked to neurodegenerative and psychiatric disorders, including Alzheimer's disease and depression [2, 3, 4]. Clinical studies indicate that patients undergoing surgical procedures are at an increased risk of memory deficits, which can progress to irreversible cognitive dysfunction. However, only about 10% of postoperative patients experience cognitive impairment lasting up to 3 months, suggesting that many maintain normal cognitive function despite the stress of surgery or infection [5]. Similarly, in Alzheimer's disease, a prolonged preclinical phase characterized by asymptomatic pathological changes and the accumulation of central inflammatory cytokines occurs before cognitive decline [6], indicating the presence of cognitive resilience before clinical symptoms emerge and that neurobiological factors inherent to cognitive function may underlie this resilience following cognitive impairments.

Systemic inflammation significantly contributes to cognitive deficits, particularly in conditions such as sepsis‐associated delirium (SAD) and postoperative delirium (POD). Research has demonstrated that inflammation‐induced deficits in hippocampus‐dependent working memory are primarily mediated by circulating IL‐1β rather than locally synthesized IL‐1β within the hippocampus [7]. Systemic administration of IL‐1β is known to impair learning and memory, primarily by affecting hippocampal pyramidal neurons and disrupting synaptic plasticity [8]. The use of IL‐1 receptor antagonists (IL‐1Ra) has been shown to alleviate these electrophysiological changes [9, 10], emphasizing the importance of maintaining homeostatic hippocampal activity to prevent learning and memory deficits. The impact of IL‐1β on cognitive function is influenced by various factors, including mental health status, types of injuries, and trauma severity [11]. Notably, some individuals display cognitive resilience in response to inflammation‐induced stress [12]. The decline in cognitive function associated with inflammation may be reversible through IL‐1R depletion, suggesting that the IL‐1β signaling pathway is involved in cognitive impairments [13]. However, further research is needed to clarify the behavioral implications of IL‐1β‐induced cognitive variations and their underlying mechanisms.

Earlier preclinical studies suggest that some individuals exhibit resistance to inflammation‐induced apoptosis, indicating that certain neurons or neural circuits may possess mechanisms that render them less sensitive to inflammation or immune stressors. This may modulate susceptibility to cognitive dysfunction through distinct pathways [14]. It is well‐established that cognitive resilience is influenced by factors such as overall health, lifestyle choices, comorbidities, and genetic predispositions. However, the neurobiological factors underlying cognitive resilience in the context of systemic inflammation remain poorly understood.

In this study, we propose that hippocampal pyramidal neurons play a crucial role in promoting cognitive resilience to systemic inflammation by maintaining balanced neural activity in a region‐specific manner. Our findings indicate that IL‐1β‐induced systemic inflammation impairs cognitive flexibility, which is essential for adapting responses to changing environments based on mnemonic discrimination. Importantly, this impairment does not affect the learning process or memory formation. This effect was specifically observed during the reversal stage of the Barnes maze test (BMT) in male mice. Subsequent experiments demonstrated that the activity of dorsal CA3 (dCA3) pyramidal neurons is crucial for regulating cognitive resilience against behavioral inflexibility, especially under adverse conditions. Together, our data underscore the importance of the dorsal CA3 (dCA3) region in maintaining cognitive balance during context‐dependent stress. These findings provide novel insights into the neural substrates underlying cognitive flexibility in the context of inflammation.

## 2 Materials and Methods

### 2.1 Animals

Male or female C57BL/6J mice, aged 3–4 months, were obtained from Vital River (Beijing, China) and housed in groups of five at 22°C with a 12‐h light/dark cycle, with food and water provided ad libitum. All experimental procedures were conducted in accordance with the guidelines approved by the Air Force Medical University Animal Center. Prior to behavioral testing, the mice were handled daily for 3 min. Systemic inflammation was induced by administering IL‐1β intraperitoneally (i.p.) at a dose of 1 μg/kg, with 0.1% bovine serum albumin in phosphate‐buffered saline as the vehicle, 3 h before behavioral assessments [15].

### 2.2 Viral Vectors and Stereotaxic Surgeries

The AAV2/9‐CaMKII‐cre‐EYFP‐WPRE‐pA, AAV2/9‐hSyn‐DIO‐hM3Dq‐mCherry‐WPRE‐pA, AAV2/9‐hSyn‐DIO‐hM4Di‐mCherry‐WPRE‐pA, AAV2/9‐hSyn‐CaMKII‐GCaMP7s‐WPRE‐pA, and AAV2/9‐CaMKII‐ChR2‐mCherry‐WPRE‐pA (at a concentration of 10^13^ viral particles/mL, stored in aliquots at −80°C) were purchased from Taitool (Shanghai, China). For stereotaxic injections, mice were anesthetized with 2% isoflurane and secured in a stereotactic apparatus (Stoelting, USA). After hair removal, the skull was exposed and cleaned with 0.9% saline. Small perforations were made in the skull using a drill, and approximately 200 nL of virus was injected into specific brain regions at a rate of 60–80 nL/min using a pulled glass capillary (Narishige PC‐100, Japan). The micropipette was left in each injection site for 10 min before withdrawal. Referring to *The Paxinos & Franklin Mouse Brain in Stereotaxic Coordinates* (5th edition), the coordinates for dCA3 were set as AP: −1.67 mm, ML: ±1.55 mm, and DV: −2.35 mm, and for DG as AP: −1.67 mm, ML: ±0.75 mm, and DV: −2.32 mm.

### 2.3 Open Field Test

Before behavioral testing, mice were given a 30‐min habituation period in the testing environment. The open field apparatus consisted of a square area measuring 40 × 40 cm, segmented into 8 × 8 cm squares. Mice were placed in the open field for a 5‐min session, during which their total distance traveled and time spent in the center zone were recorded to assess locomotor activity and anxiety levels, respectively. After each trial, the apparatus was cleaned with 75% alcohol to eliminate any residual animal odors.

### 2.4 Barnes Maze Test

The Barnes maze experiments were conducted in an environment with 80 dB white noise and 120 W light. During the acquisition stage spanning days 1 to 4, 3 h post‐intraperitoneal injection of IL‐1β, mice were given timed sessions to explore the platform for up to 3 min until they successfully reached the escape cage (or were gently guided if they did not locate the hole) for two consecutive trials per day. A total of eight trials were conducted to prevent the influence of overly repeated training on the results.

On day 5 (probe trial), the escape hole was removed, and mice had a 2‐min exploration period on the platform. Subsequently, during the reversal stage, the previous escape cage was relocated to the opposite zone, and mice were allowed to explore the platform until they entered this new cage (reversal hole).

Latency to the escape hole and time spent in the target zone were utilized to evaluate spatial cognitive function. Mice whose performance fell below a predetermined cut‐off were categorized as “cognitively susceptible,” while those surpassing the cut‐off were deemed “cognitively resilient.” The cut‐off value was defined as the mean plus one SD for the control group. Following each trial, the platform was cleaned with 75% alcohol. All experimental procedures were monitored and analyzed using the ANY‐maze software (Stoelting, USA).

### 2.5 Fiber Photometry

In the in vivo fiber photometry procedure, animals were administered AAV2/9‐CaMKII‐GCaMP7s‐WPRE‐pA in the dorsal CA3 region. An optical fiber (200 μm in diameter, 3.5 mm in length, NA 0.37, Inper, China) was surgically implanted in the dorsal CA3 region as previously described [16]. Throughout the behavioral tasks, animal tracking videos and in vivo Ca^2+^ signals were continuously monitored.

The Ca^2+^ signals were captured using a fiber photometry system from Thinker Tech Nanjing Bioscience Inc. Subsequently, the data of Ca^2+^ signals were analyzed using custom‐written MATLAB scripts (MATLAB, MathWorks). The responses of GcaMP7s‐infected neurons were calculated as the Z‐score of *ΔF*/*F*, defined as (*v*_signal_‐*F*_0_) /STD (*v*_signal_), where *F*_0_ represented the mean fluorescence signal. For the trace and heatmap of calcium signals, each data point represents an individual mouse, with data presented as mean ± SEM of signal. Time point (labeled as 0 s) corresponds to the start time point of behavioral events.

### 2.6 Electrophysiology

Mice were sacrificed under isoflurane anesthesia, and then brain was removed after cognitive tests, ensuring minimal time above the ice. Brain sections (300 μm thick), including dorsal hippocampus, were harvested in an iced cutting solution (containing NMDG, NaCl, KCl, HEPES, glucose, NaHCO_3_, NaH_2_PO_4_, ascorbate, pyruvate, thiourea, MgSO_4_, CaCl_2_) using a vibratome (VT1200S, Leica). The sections were then transferred into a 36°C oxygenated holding solution (composed of NaCl, KCl, HEPES, glucose, NaHCO_3_, NaH_2_PO_4_, ascorbate, pyruvate, thiourea, MgSO_4_, CaCl_2_) for 1 h, followed by immersion in a recording chamber filled with ACSF (NaCl, KCl, HEPES, glucose, NaHCO_3_, NaH_2_PO_4_, MgSO_4_, CaCl_2_). Whole‐cell recordings were conducted for all experiments. Pipettes were pulled to a resistance of 4–10 MΩ using a P97 puller (Sutter Instruments, USA). Signal recordings were carried out with a 700B amplifier and 1.3 kHz filter. All the recording data were analyzed using Clampfit 10.9 software (Molecular Devices, Sunnyvale, CA, USA). The intracellular solution used contained (130 mM potassium gluconate, 4 mM KCl, 10 mM HEPES, 0.3 mM EGTA, 10 mM Na_2_‐phosphocreatine, 4 mM Mg_2_‐ATP, 0.3 mM Na_2_‐GTP, pH 7.4, 290–300 mOsm). For action potential recordings, currents were injected into neuron, followed by perfusion with CNO (1 μM) to confirm the correct expression of the chemogenetic virus.

### 2.7 Immunofluorescence

Mice were anesthetized with isoflurane and transcardially perfused with 0.9% normal saline, followed by 4% paraformaldehyde (PFA) for fixation. Subsequently, the brains were extracted and postfixed in 4% PFA for 4 h, then transferred to a 30% sucrose solution until they sank. Brain sections were sliced at a thickness of 35 μm, treated with a 10% donkey serum solution (supplemented with 0.3% Triton X‐100) for 30 min, and then incubated overnight at 4°C with primary antibodies diluted in an antibody dilution solution. The primary antibodies used in the experiments included CaMKII (CST, 50049S), GABA (Genetex, GTX125988), and c‐Fos (Synaptic Systems, 226,308). After incubation, the sections were washed three times with PBS and then exposed to secondary antibodies Alexa Fluor 488, 594, and 647 (Abcam) at a dilution of 1:500 for 2 h at room temperature. Following another three washes with PBS, the sections were mounted on glass coverslips using mounting media containing DAPI (ab104139, Abcam). Subsequently, the sections were imaged using a confocal microscope FV1200, and the images were analyzed using ImageJ software.

### 2.8 Blood‐brain Barrier Disruption Evaluation

IL‐1β or vehicle was administered via intraperitoneal injection for 5 consecutive days. Under isoflurane anesthesia, 2% Evans Blue (4 mL/kg, diluted in normal saline) was introduced through a 22‐G catheter inserted into the tail vein. One day after the injection, the brain, liver, lung, kidney, and spleen were harvested for analysis.

### 2.9 Statistics

All experiments were analyzed using GraphPad Prism 8. An unpaired Student's *t*‐test was employed to compare two individual groups. For comparison among more than two groups, an ordinary one‐way ANOVA or two‐way ANOVA followed by post hoc Tukey's multiple comparisons test was employed. Additionally, differences in the Barnes Maze training stage were assessed using a repeated‐measures (rm) two‐way ANOVA followed by Sidak's multiple comparisons test. Non‐normally distributed data were analyzed using the Mann–Whitney test for comparisons between two groups and the Kruskal–Wallis test for comparisons among three or more groups. Proportional data were analyzed using Fisher's exact test or the χ^2^ test. Statistical significance was denoted as ****p < 0.0001, ***p < 0.001, **p < 0.01, and *p < 0.05. All data were presented as the mean ± SEM.

## 3 Results

### 3.1 IL‐1β‐Induced Systemic Inflammation Causes Cognitive Inflexibility

**FIGURE 1:**
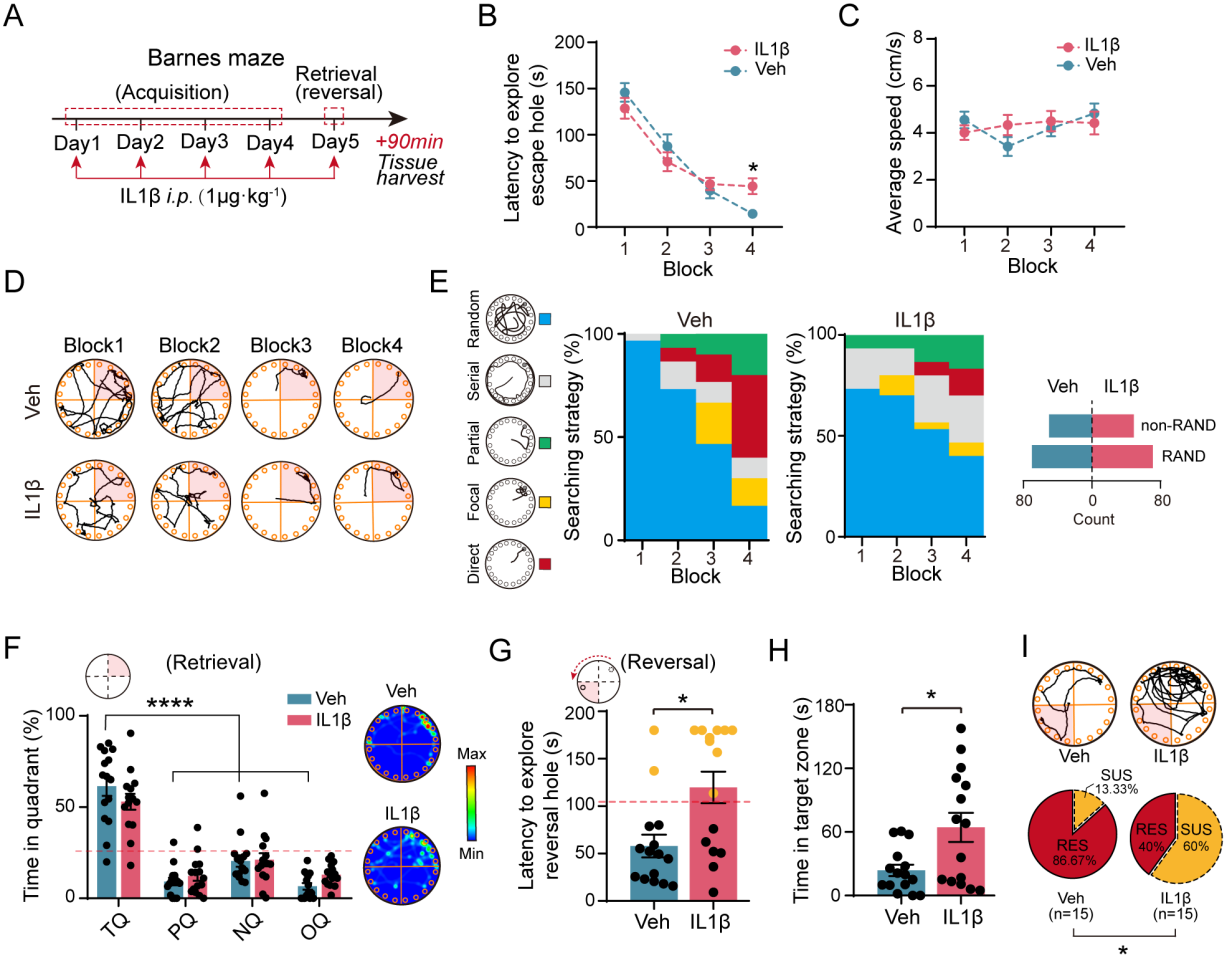
Systemic exposure to IL‐1β leads to deterioration in cognitive flexibility in male mice. (A) Schematic representation of the experimental timeline. (B) Latency to explore the escape hole during memory acquisition (rm Two‐way ANOVA, Group: *F*_(1,28)_ = 0.0111, *p* = 0.9165). (C) Average speed during memory acquisition (rm Two‐way ANOVA, Group: *F*_(1,28)_ = 0.0226, *p* = 0.8816). (D) Behavioral track plots during memory acquisition for vehicle‐ and IL‐1β‐treated male mice. (E) Schematic illustration of different searching strategies (left panel) and analysis of searching strategy in male mice exposed to IL‐1β or Vehicle during acquisition (right panel) (Fisher's exact test, *p* = 0.99). (F) Time spent (%) in different quadrants and sample track heatmaps of vehicle‐ and IL‐1β‐treated male mice during probe trial. TQ, target quadrant, PQ, positive quadrant, NQ, negative quadrant, OQ, opposite quadrant (Two‐way ANOVA, Group: *F*_(1,112)_ = 0.0292, *p* = 0.8646). (G) Latency to reach the reversal hole was measured in vehicle‐ and IL‐1β‐treated male mice during the BMT. The red dashed line indicates the cut‐off value, calculated as the mean plus one standard deviation (SD) (Mann–Whitney test, *p* = 0.013). (H) Time (s) spent in the target zone of vehicle‐ and IL‐1β‐treated male mice during the reversal stage of BMT (Mann–Whitney test, *p* = 0.036). (I) Track plots of the vehicle or IL‐1β groups during reversal stage (top panel), along with proportion of resilient and susceptible male mice (below panel) (Fisher's exact test, *p* = 0.0209). *n* = 15 mice per group. *p < 0.05; **p < 0.01; ****p < 0.0001. All values are presented as mean ± SEM.

To characterize cognitive dysfunction influenced by systemic inflammation, we conducted the BMT on both male and female mice that received intraperitoneal injections of IL‐1β 3 h prior to training or testing over five consecutive days (Figure 1A). Our results indicated that a single administration of IL‐1β did not affect the animals' overall exploration behavior or anxiety levels in the open field test (OFT), as evidenced by similar total distances traveled and time spent in the center zone between the vehicle and IL‐1β‐treated groups. Furthermore, five consecutive days of IL‐1β exposure did not produce any discernible changes in total distance traveled or time spent in the center zone during the OFT (Figure S1A–E). Additionally, Evans blue staining results indicated that consecutive IL‐1β injections did not compromise blood–brain barrier integrity (Figure S1F).

In the BMT, our results revealed no significant differences in the latency to explore the escape hole or in average speed between male or female mice treated with IL‐1β and corresponding controls administered the vehicle during the training trials (Figure 1B–D and Figure S2B–D). Although IL‐1β‐exposed mice demonstrated cognitive performance similar to that of control mice during testing, notable differences emerged in the efficiency and strategies used to locate the escape hole. Specifically, IL‐1β‐treated male or female mice tended to adopt a non‐spatial strategy (random) on day 4, whereas vehicle‐treated mice exhibited a clear preference for spatial strategies (serial, partial, focal, accurate) (Figure 1E and Figure S2E). Furthermore, during the retrieval phase, IL‐1β‐treated mice spent more time in the target zone compared to non‐spatially associated zones (positive, negative, and opposite). Similar trends were observed in vehicle‐treated animals (Figure 1F and Figure S2F), indicating that while IL‐1β did not directly impair memory acquisition and retrieval, it affected spatial learning efficiency.

To further elucidate the capacity for rapid re‐encoding of new spatial information, specifically cognitive flexibility, we conducted a transfer experiment by relocating the escape hole (initially in the target zone) to the opposite zone, referred to as the reversal hole. In this scenario, IL‐1β‐treated male mice exhibited longer search times to find the reversal hole and spent significantly more time in the original target quadrant compared to control mice (Figure 1G,H). Notably, 60% (9 out of 15) of IL‐1β‐treated male mice showed a decline in their ability to locate the reversal hole, a condition we termed “susceptible.” Conversely, the remaining 40% (6 out of 15) of male mice demonstrated resilience to the inflexibility induced by IL‐1β injection and were classified as the “resilient” group. This was distinctly different from the vehicle‐treated control group, where only 13.4% of the mice were susceptible, while 86.6% demonstrated resilience (Figure 1I).

In contrast, treatment with IL‐1β did not impair cognitive flexibility in female mice. Specifically, during the reversal stage, IL‐1β‐treated female mice spent a similar amount of time locating the reversal hole as vehicle‐treated control female mice (Figure S2H,I). Moreover, the percentages of “susceptible” and “resilient” female mice following either vehicle or IL‐1β administration were comparable between the two groups (Figure S2J). These findings indicate that IL‐1β exposure disrupts the ability to flexibly re‐encode spatial information in male mice but not in female mice, highlighting a sexually dimorphic response in rodents regarding cognitive function under inflammatory conditions. Based on these results, we focused on examining the effects of IL‐1β on cognitive flexibility exclusively in male mice in the subsequent experiments.

### 3.2 dCA3 Neuron Activation is Involved in Maintaining the Cognitive Resilience to Systemic Inflammation

**FIGURE 2:**
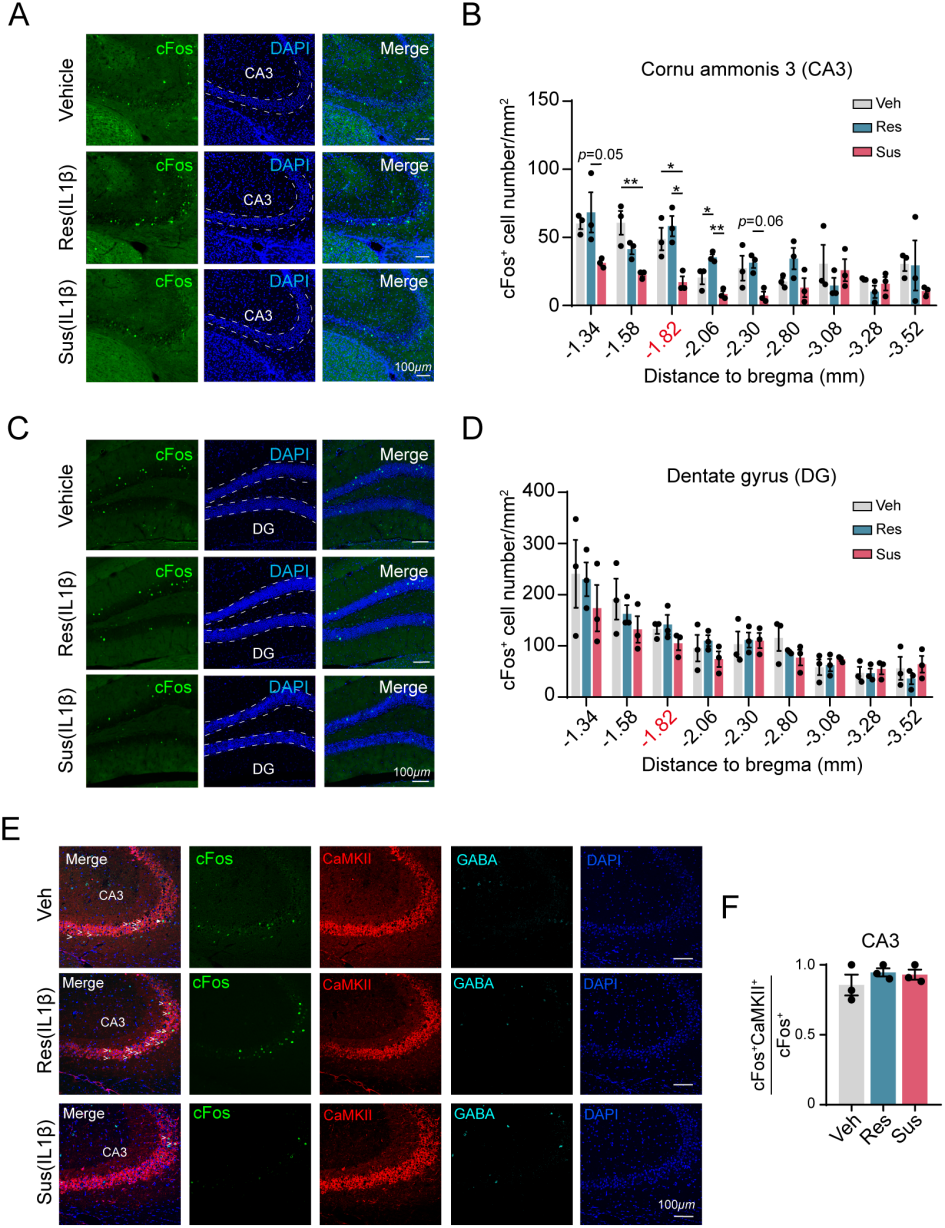
c‐Fos expression profiling in hippocampal subfields of cognitively resilient and susceptible mice. (A) Representative images of c‐Fos expression in CA3 subfield (1.82 mm posterior to bregma) for vehicle, resilient, and susceptible groups. DAPI with blue, and c‐Fos with green. (B) Statistical analysis of density of c‐Fos + cell in CA3 (Two‐way ANOVA, Group: *F*_(2,54)_ = 19.46, *p* < 0.0001). (C) Representative images of c‐Fos expression in DG subfield (1.82 mm posterior to bregma) for vehicle, resilient, and susceptible groups. DAPI with blue, and c‐Fos with green. (D) Statistical analysis of density of c‐Fos^+^ cell in DG (Two‐way ANOVA, Group: *F*_(2,54)_ = 1.603, *p* = 0.2107). (E) Representative immunostaining of c‐Fos (green), CaMKII (red), GABA (Cyanine), and DAPI (blue). (F) Analysis of colocalization between c‐Fos and CaMKII of CA3 in vehicle, resilient, and susceptible groups (One‐way ANOVA, *F*_(2,6)_ = 0.8999, *p* = 0.4552). Scale bar: 100 μm. *n* = 3 mice per group. *p < 0.05; **p < 0.01. All values are presented as mean ± SEM.

To explore the mechanisms underlying cognitive resilience in mice exposed to IL‐1β, we harvested brain tissue 90 min after the exploration stage of the reversal task, as illustrated in Figure 1A, and performed c‐Fos staining. Our data revealed a higher density of c‐Fos^+^ cells in the dorsal hippocampal CA3 region of cognitively resilient mice compared to susceptible mice (Figure 2A,B). In contrast, the number of c‐Fos^+^ cells in the dentate gyrus (DG) was similar between the resilient and susceptible groups (Figure 2C,D), suggesting that increased neuronal activity in the dCA3 region may contribute to cognitive resilience during IL‐1β exposure. Notably, no significant differences in c‐Fos^+^ cell density were observed in the ventral hippocampus among the vehicle‐treated, IL‐1β‐treated resilient, and susceptible groups (Figure 2B–D), indicating that the ventral hippocampus likely does not play a role in regulating spatial cognitive function during IL‐1β administration.

We next sought to determine the specific cell types within the dorsal hippocampus that contribute to the regulation of cognitive resilience. Immunostaining for c‐Fos, CaMKII, and GABA was performed in the hippocampal region. Our analysis revealed that over 90% of activated neurons (c‐Fos^+^ neurons) in both the dCA3 and DG regions were identified as pyramidal neurons rather than interneurons (Figure 2E,F and Figure S4A,B). Furthermore, we observed no significant differences in the colocalization of c‐Fos^+^ neurons with pyramidal neurons between the susceptible and resilient groups (Figure 2E,F).

**FIGURE 3:**
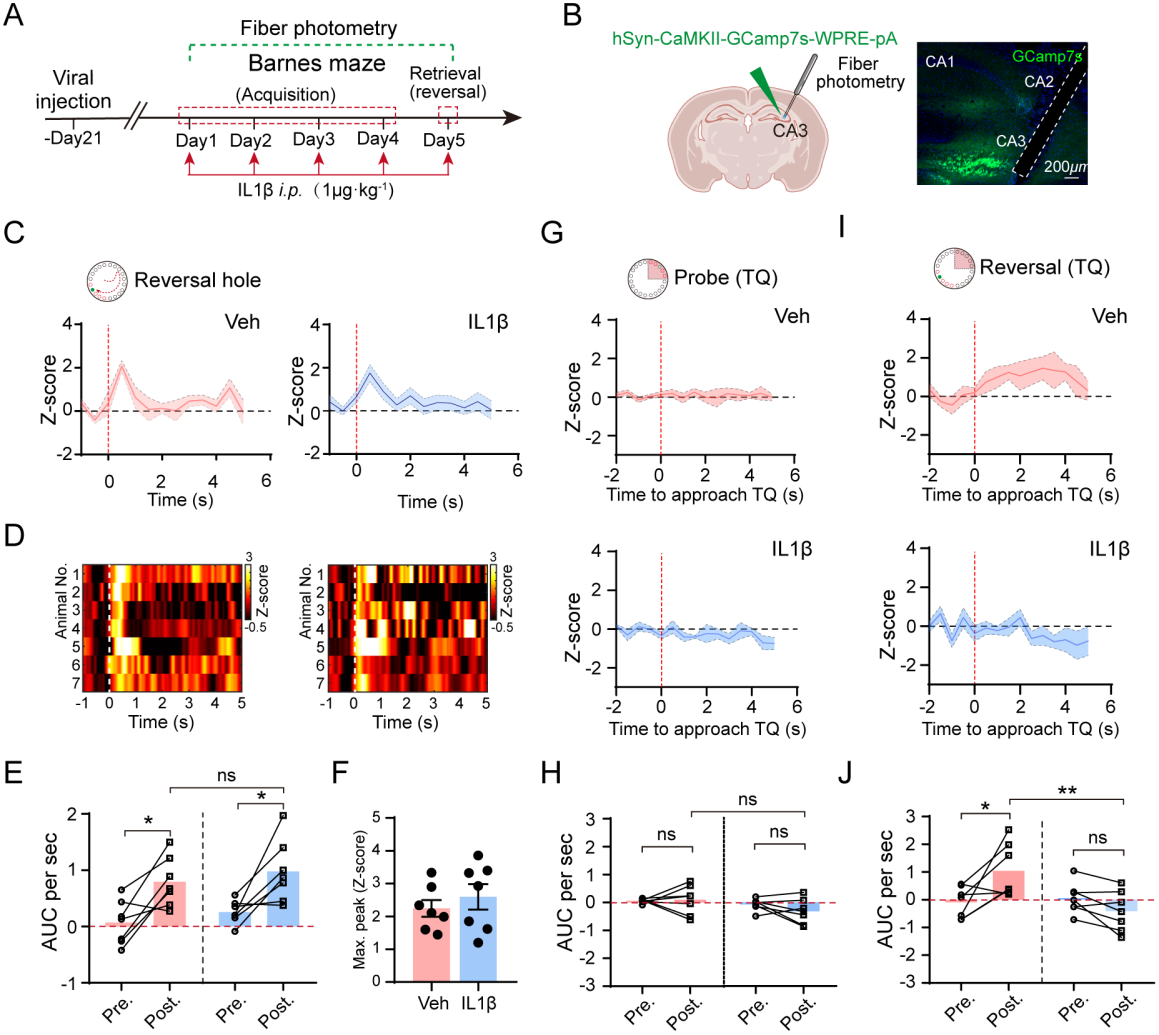
Neuronal response patterns of dCA3 CaMKII^+^ neurons in IL‐1β‐treated mice during BMT performance. (A) Schematic representation of the experiment timeline. (B) Illustration and verification of the location of viral injection and optical fiber implantation. Created with Biorender.com. Scale bar: 200 μm. (C, D) Traces and heatmaps of dCA3 CaMKII^+^ neuronal responses during the reversal test of BMT in the vehicle and IL‐1β groups. Red and white dashed lines indicate the time point when the animal enters the reversal hole. (E) During reversal stage, areas under the Z‐score curve (AUC) of averaged neuronal activity traces before and during exploration of the reversal hole in vehicle and IL‐1β groups (Two‐way ANOVA, Group: *F*_(1,24)_ = 1.368, *p* = 0.2537). (F) Maximal peak (Z‐score) of neuronal activity in vehicle and IL‐1β groups when the animals entering the reversal hole (Unpaired student's *t*‐test, *p* = 0.4646). (G) Z‐scored neuronal activity traces of vehicle‐ and IL‐1β‐treated mice before and during approaching the target zone in the probe trial of BMT. (H) Quantification of AUC per second of Z‐scored average neuronal activity traces of vehicle‐ and IL‐1β‐treated mice before and during approaching the target zone in the probe trial of BMT (Two‐way ANOVA, Group: *F*_(1,24)_ = 3.971, *p* = 0.0578). (I) Z‐scored neuronal activity traces of vehicle‐ and IL‐1β‐treated mice before and during approaching the previous target zone in the reversal stage of BMT. (J) Quantification of AUC per second of Z‐scored average neuronal activity traces of vehicle‐ and IL‐1β‐treated mice before and during approaching the previous target zone in the reversal stage of BMT (Two‐way ANOVA, Group: *F*_(1,24)_ = 5.951, *p* = 0.0225). *n* = 7 mice per group. *p < 0.05; **p < 0.01. All values are presented as mean ± SEM.

How is neuronal activity altered during IL‐1β exposure? We administered AAV2/9‐CaMKII‐DIO‐GCaMP7s‐WPRE‐pA virus into the dCA3 region of adult mice and allowed for surgical recovery. Subsequently, we conducted photometry experiments (Figure 3A,B). Our findings revealed that following IL‐1β administration, there was no significant difference in the activity of CaMKII^+^ neurons compared to vehicle mice while exploring the reversal hole, as indicated by an increase in the area under the curve (Z‐score) per second when the mice found the reversal hole in both groups (Figure 3C–E). Additionally, the maximum peak (Z‐score) was comparable between the vehicle and IL‐1β‐treated mice (Figure 3F). Interestingly, during the probe trial, responses to approaching the target zone were similar between the two groups (Figure 3H). However, during the reversal stage, exposure to IL‐1β inhibited the activity of CaMKII^+^ neurons as the mice approached the previous target quadrant (Figure 3I,J), suggesting that the activation of dCA3 CaMKII^+^ neurons plays a role in mitigating cognitive inflexibility triggered by systemic inflammation.

### 3.3 Engagement of Hippocampal dCA3 CaMKII ^+^ Neuron Promotes Cognitive Resilience against IL‐1β‐Induced Memory Deficits

**FIGURE 4:**
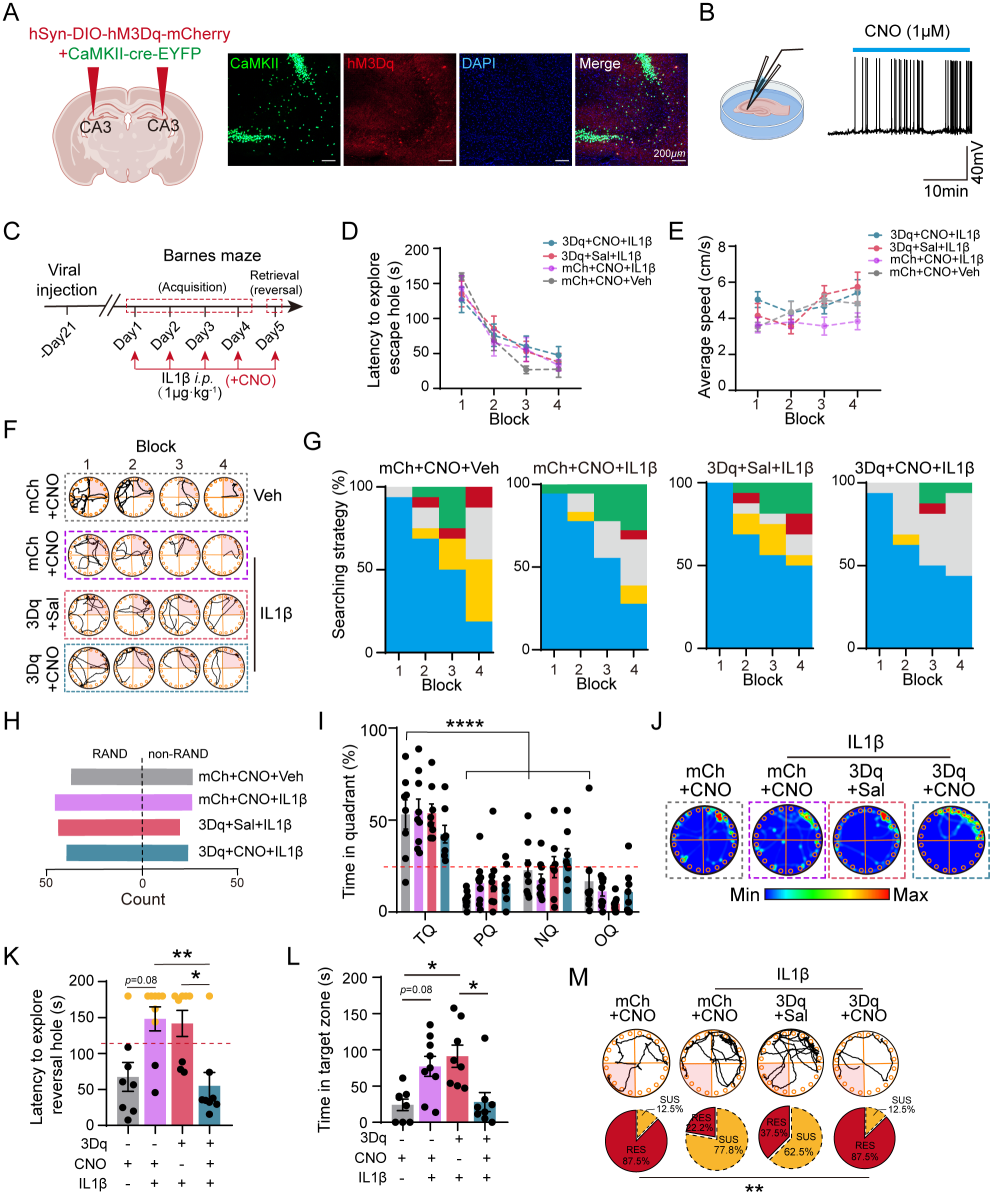
Chemogenetic activation of dCA3 CaMKII^+^ neurons ameliorates cognitive inflexibility induced by systemic inflammation. (A) Representative images of coronal sections of hippocampus showing dCA3 labeled with CaMKII‐Cre (green) dependent hM3Dq expression (mCherry) and stained with DAPI (blue). (B) Representative image of ex vivo recording and electrophysiological trace shows application of CNO (1 μM) on hM3Dq‐infected dCA3 neuron enhancing neural spiking. Created with Biorender.com. (C) Schematic diagram showing the experiment timeline of Barnes maze test for mCherry‐ or hM3Dq‐injected mice exposed to IL‐1β administration. (D) Latency to explore the escape hole during memory acquisition phase of BMT (rm Two‐way ANOVA, Group: *F*_(3,29)_ = 0.1317, *p* = 0.9405). (E) Average speed to explore the platform during memory acquisition phase of BMT (rm Two‐way ANOVA, Group: *F*_(3,29)_ = 2.226, *p* = 0.1064). (F) Track plots of BMT behavioral test in representative mice across different experimental groups during memory acquisition. (G) Quantification of different searching strategies during memory acquisition phase of BMT. (H) Comparison of non‐random and random searching strategies during acquisition phase of BMT across different experimental groups (χ^2^ test, *p* = 0.6425). (I) Percentage of time spent in different quadrants across different experimental groups (Two‐way ANOVA, Group, *F*_(3,116)_ = 0.0051, *p* = 0.995). (J) Heatmaps of representative mice in the probe trial of BMT across different experimental groups. (K) Latency to reach the reversal hole was measured during the reversal stage of BMT. The red dashed line indicates the cut‐off value, calculated as the mean plus one standard deviation (SD) (Kruskal–Wallis test, *H* = 13.66). (L) Time in the target zone across different experimental groups during the reversal stage of BMT (Kruskal–Wallis test, *H* = 14.73). (M) Track plots (up) across different experimental groups during the reversal stage of BMT and illustration of the proportion of resilient and susceptible mice (below) (χ^2^ test, *p* = 0.0081). *n* = 8 mice for mCh + CNO + Veh group; *n* = 9 mice for mCh + CNO + IL‐1β group; *n* = 8 mice for 3Dq + Sal + IL‐1β group; *n* = 8 mice for 3Dq + CNO + IL‐1β group. *p < 0.05; **p < 0.01; ****p < 0.0001. All values are presented as mean ± SEM.

To investigate the role of hippocampal dCA3 CaMKII^+^ neurons in modulating the rapid re‐encoding of spatial information under systemic inflammation, we employed chemogenetic stimulation using designer receptors exclusively activated by designer drugs (DREADDs). We performed bilateral stereotaxic injections of AAV2/9‐CaMKII‐Cre‐EYFP‐WPRE‐pA along with either AAV2/9‐hSyn‐DIO‐hM3Dq‐mCherry‐WPRE‐pA or AAV2/9‐hSyn‐DIO‐mCherry‐WPRE‐pA into the dorsal CA3 subfield (Figure 4A). Ex vivo recordings demonstrated that perfusion of clozapine‐N‐oxide (CNO), the pharmacological ligand for DREADDs, onto dCA3 CaMKII^+^ neurons resulted in a significant increase in their firing frequency (Figure 4B).

The mice were housed in standard cages for 3 weeks to allow for surgical recovery and viral expression. CNO or saline was administered intraperitoneally 30 min prior to each daily behavioral training or testing session (Figure 4C). Activation of dCA3 CaMKII^+^ neurons by CNO resulted in an overall increase in total exploration in the OFT; however, there was no significant difference in the time spent in the center zone compared to the control groups, indicating that dorsal CA3 CaMKII^+^ neuron activation did not influence anxiety behavior (Figure S5A,B). Notably, CNO did not affect the exploratory behavior of mice injected with the control vector AAV2/9‐hSyn‐DIO‐mCherry‐WPRE‐pA (Figure S5B), underscoring the specificity of DREADD activation by CNO in our experimental approach.

We then investigated whether the activation of dCA3 CaMKII^+^ neurons modulates cognitive flexibility in the context of inflammation. Stimulation of hippocampal dCA3 CaMKII^+^ neurons with CNO did not affect the exploration latency of mice in locating the escape hole compared to the IL‐1β + mCherry + CNO group. Similarly, there was no significant difference in exploration latency between the IL‐1β + hM3Dq + Sal and IL‐1β + hM3Dq + CNO groups (Figure 4D–F), indicating that the activation of dCA3 CaMKII^+^ neurons did not impact memory acquisition. Validation of average speed suggested intact locomotor activity in the behavioral memory test (BMT) (Figure 4E). Interestingly, we observed a distinct exploration strategy in the IL‐1β + hM3Dq + CNO group compared to the IL‐1β + hM3Dq + Sal group. Activation of dCA3 CaMKII^+^ neurons in mice exposed to IL‐1β led to a shift toward a serial searching strategy, unlike the saline‐treated group (Figure 4G,H). This suggests that increased dCA3 CaMKII^+^ neuronal activity may enhance exploratory motivation to locate the escape hole.

In the retrieval stage, we observed that the activation of dCA3 CaMKII^+^ neurons did not affect the time spent in the target quadrant. All three groups of mice spent longer durations in the target quadrant compared to the other quadrants. Importantly, no significant difference was found between the IL‐1β + hM3Dq + Sal and IL‐1β + mCherry + CNO groups (Figure 4I,J), ruling out the possibility of off‐target effects induced by CNO.

During the reversal stage, where the updated location of the escape hole was used to assess memory flexibility, we noted that activation of dCA3 CaMKII^+^ neurons mitigated the memory flexibility deficits induced by consecutive IL‐1β administrations compared to the IL‐1β + hM3Dq + Sal group. This improvement was evidenced by a reduced latency to find the reversal hole (Figure 4K) and decreased time spent in the previous target quadrant (Figure 4L) in the IL‐1β + hM3Dq + CNO mice. Notably, we observed a significant decrease in the proportion of cognitively susceptible mice in the IL‐1β + hM3Dq + CNO group (12.5% susceptible, 87.5% resilient) compared to the IL‐1β + hM3Dq + Sal group (62.5% susceptible, 37.5% resilient) (Figure 4M).

### 3.4 Inhibition of CaMKII ^+^ Neuron Mimics Cognitive Inflexibility Induced by IL‐1β Injection

**FIGURE 5:**
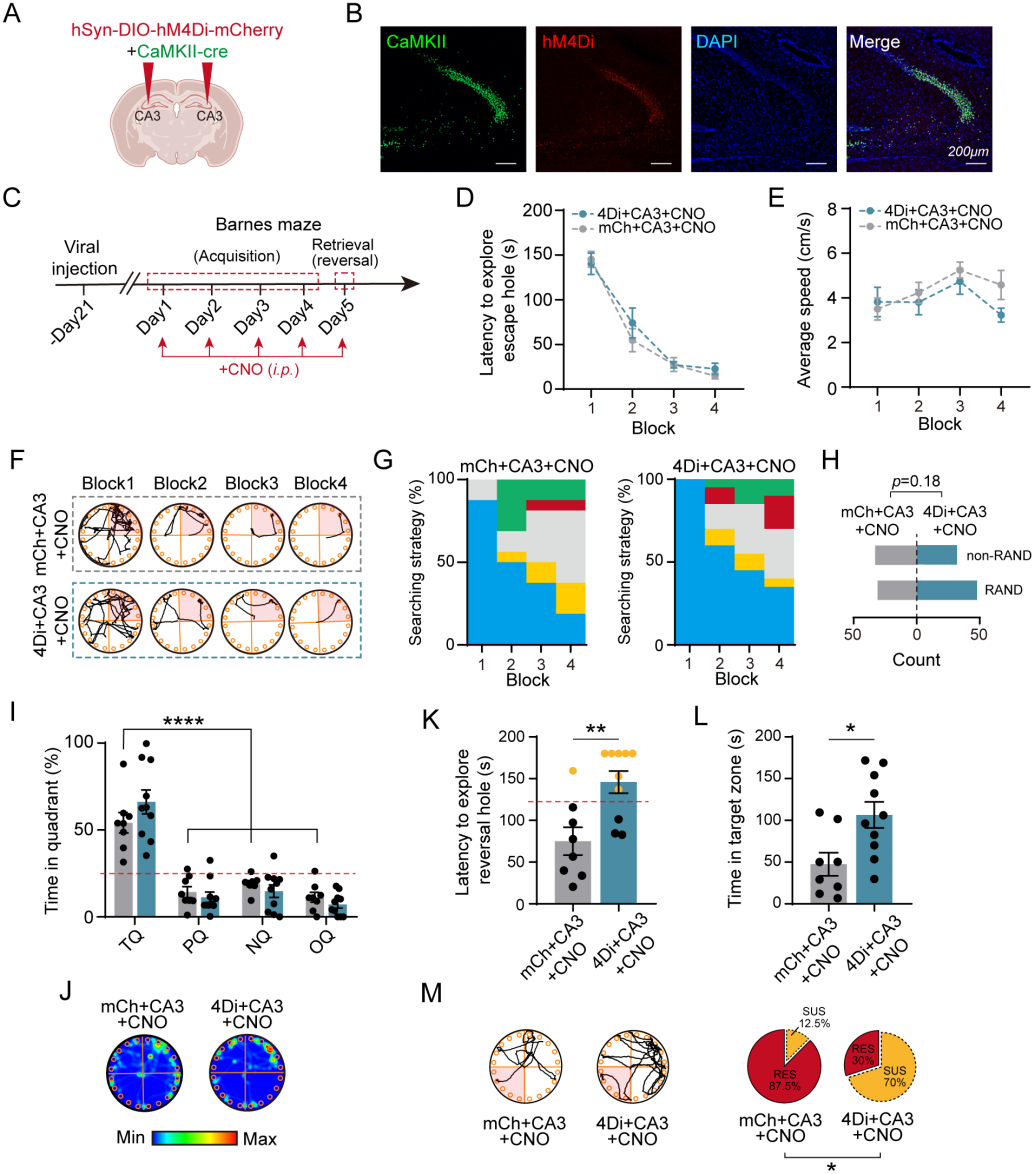
Chemogenetic inhibition of dCA3 CaMKII^+^ neurons attenuates cognitive flexibility. (A) Schematic representation of bilateral viral injection in dCA3. Created with Biorender.com. (B) Representative images of CaMKII‐Cre (green) dependent hM4Di expression (red) in CA3 and stained with DAPI (blue). (C) Schematic diagram showing the experiment timeline of Barnes maze test for mCherry‐ or hM4Di‐injected mice treated with CNO. (D) Latency to explore the escape hole during memory acquisition phase of BMT (rm Two‐way ANOVA, Group: *F*_(1,16)_ = 0.3278, *p* = 0.5749). (E) Average speed to explore the platform during memory acquisition phase of BMT (rm Two‐way ANOVA, Group: *F*_(1,16)_ = 0.8942, *p* = 0.3584). (F) Track plots of BMT behavioral test for mCherry‐ or hM4Di‐injected mice treated with CNO during memory acquisition. (G) Analysis of different searching strategies during memory acquisition phase of BMT. (H) Comparison of non‐random and random searching strategies during acquisition phase of BMT for mCherry‐ or hM4Di‐injected mice treated with CNO (Fisher's exact test, *p* = 0.18). (I) Percentage of time spent in different quadrants for mCherry‐ or hM4Di‐injected mice treated with CNO (Two‐way ANOVA, Group: *F*_(1,64)_ = 0.004, *p* = 0.9492), (J) Heatmaps of representative mice in the probe trial of BMT for mCherry‐ or hM4Di‐injected mice treated with CNO. (K) Latency to reach the reversal hole during the reversal stage of BMT. The red dashed line indicates the cut‐off value, calculated as the mean plus one SD (Mann–Whitney test, *p* = 0.0059). (L) Time in the target zone for mCherry‐ or hM4Di‐injected mice treated with CNO during the reversal stage of BMT (Unpaired student's *t*‐test, *p* = 0.014). (M) Track plots (left) of mCherry‐ or hM4Di‐injected mice treated with CNO during the reversal stage of BMT, and the proportion of resilient and susceptible mice (right) (Fisher's exact test, *p* = 0.0248). *n* = 8 mice for mCh + CA3 + CNO; *n* = 10 mice for 4Di + CA3 + CNO. Scale bar: 200 μm. *p < 0.05; **p < 0.01; ****p < 0.0001. All values are presented as mean ± SEM.

To further investigate the functional significance of dCA3 CaMKII^+^ neurons and determine whether their activation is necessary to ameliorate the abnormal memory flexibility induced by systemic inflammation, we bilaterally infused AAV2/9‐CaMKII‐Cre‐EYFP‐WPRE‐pA along with either AAV2/9‐hSyn‐DIO‐hM4Di‐mCherry‐WPRE‐pA or AAV2/9‐hSyn‐DIO‐mCherry‐WPRE‐pA viruses into the dorsal CA3 region (Figure 5A,B). Ex vivo recordings revealed a significant reduction in spiking activity of CaMKII^+^ neurons following CNO perfusion (Figure S5E). After a 3‐week recovery period post‐stereotactic injection to allow for surgical recovery and viral expression, we conducted the OFT and behavioral memory test (BMT) on the experimental subjects. Our results showed that administration of CNO in the hM4Di + CNO group did not lead to significant changes in total distance traveled or time spent in the center zone compared to the control groups (Figure S5C,D).

Throughout the training sessions (Figure 5C), inhibition of CaMKII^+^ neurons in the dCA3 region resulted in comparable exploration latencies for locating the escape hole (Figure 5D,F) and similar movement speeds (Figure 5E), indicating that suppression of dCA3 pyramidal neurons did not impede spatial memory formation. Notably, by the final day of training, mice with inhibited dCA3 neurons demonstrated a robust adaptation of spatial strategies (Figure 5G,H).

During the retrieval phase, no significant differences were observed in the time spent in the target quadrant between the mCherry + CNO and hM4Di + CNO groups, with all groups spending longer durations in the target quadrant compared to the other quadrants (Figure 5I,J). This suggests that inhibition of dCA3 pyramidal neurons did not hinder memory retrieval. However, after relocating the escape cage to the opposite quadrant, suppression of dCA3 excitatory neurons resulted in a longer duration to locate the updated escape hole and increased time spent in the previous target zone, unlike the control group (Figure 5K,L). Additionally, we observed a significant increase in the percentage of cognitively susceptible mice in the hM4Di + CNO group targeting dCA3 neurons, with 70% identified as vulnerable and 30% as resilient. In contrast, the mCherry + CNO group showed that 87.5% were resilient and only 12.5% were vulnerable (Figure 5M). These findings indicate that the specific inhibition of dCA3 pyramidal neurons impaired memory flexibility while preserving memory encoding and retrieval, highlighting the critical role of dCA3 in memory generalization.

We next aimed to investigate the specific role of dCA3 CaMKII^+^ neurons in modulating cognitive resilience. Given that the DG is another crucial hippocampal subregion involved in learning and memory [17], we sought to determine whether the DG influences cognitive flexibility in the context of inflammation. To this end, we bilaterally infused AAV2/9‐CaMKII‐Cre‐EYFP‐WPRE‐pA along with either AAV2/9‐hSyn‐DIO‐hM4Di‐mCherry‐WPRE‐pA or AAV2/9‐hSyn‐DIO‐mCherry‐WPRE‐pA into the DG region. Post‐experimental immunohistochemical staining (Figure S6A–C) and ex vivo recordings confirmed successful viral transduction and functional responses to CNO application (Figure S5F).

During the BMT training sessions, inhibition of CaMKII^+^ neurons in the DG led to delayed exploration latencies for locating the escape hole (Figure S6D,F), while movement speeds remained comparable to those of the control group (Figure S6E). This suggests that suppression of DG pyramidal neurons hindered spatial memory formation. Notably, unlike the dCA3‐inhibited group, inhibition of DG neurons increased the use of a random search strategy during training (Figure S6G,H). Furthermore, CNO administration to inhibit DG pyramidal neurons expressing inhibitory DREADDs did not affect behavioral performance during the retrieval (Figure S6I,J) or reversal phases (Figure S6K–M) compared to controls. The proportion of cognitively vulnerable mice in the hM4Di + CNO DG group (22.2% vulnerable, 77.8% resilient) was similar to that in the control group (11.1% vulnerable, 88.9% resilient) (Figure S6M right panel). These results indicate that while specific inhibition of DG pyramidal neurons impairs spatial memory encoding, it does not influence memory retrieval or cognitive adaptability. This underscores the critical role of dCA3 pyramidal neurons in modulating cognitive flexibility, particularly in the context of inflammation.

### 3.5 Hippocampal dCA3 CaMKII ^+^ Neurons Construct a Network Modulating Cognitive Function

**FIGURE 6:**
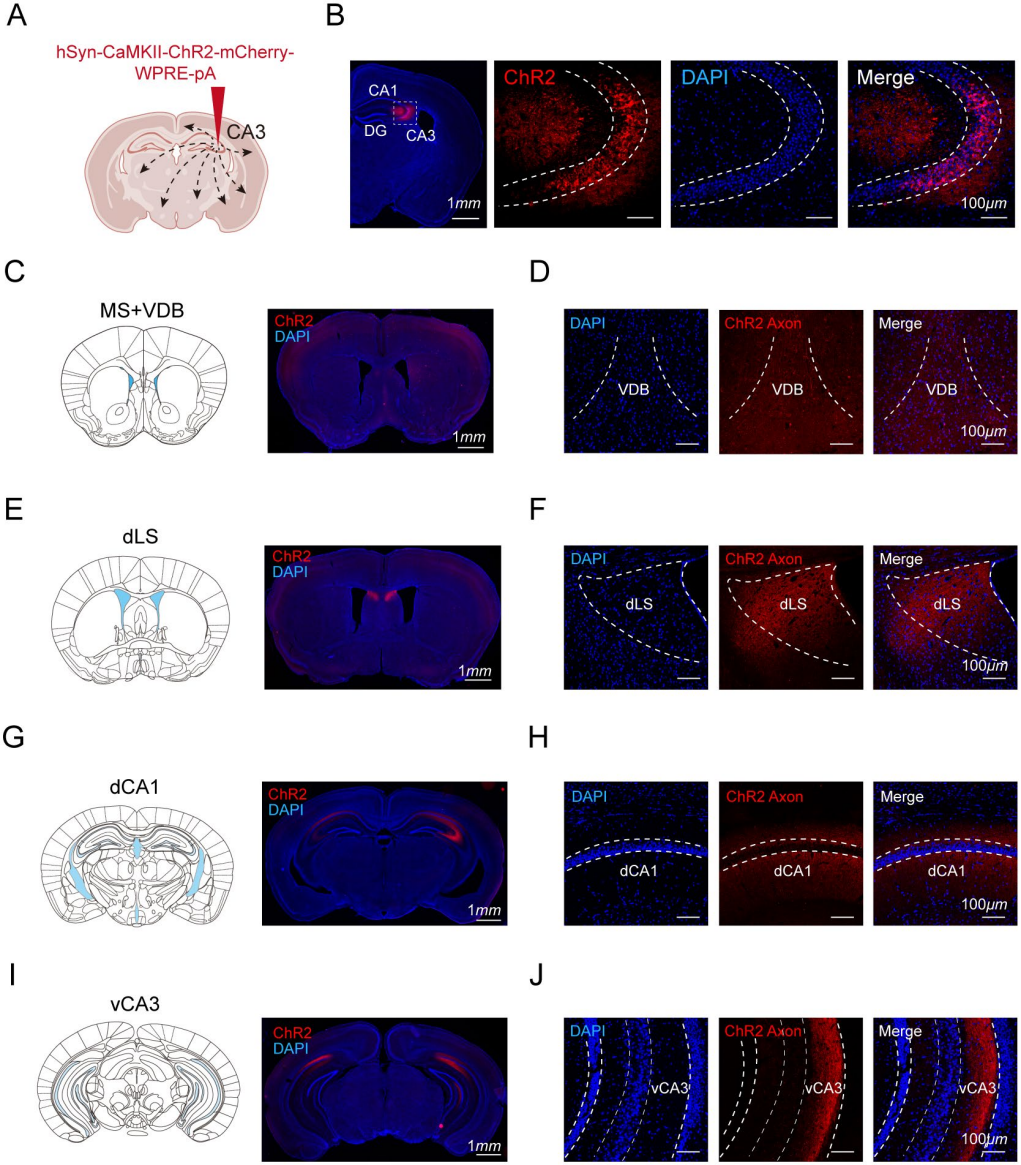
Efferents from the hippocampal dCA3 form a neural network involved in cognitive processes. (A) Schematic representation of CaMKII‐ChR2 injection in dCA3. Created with Biorender.com. (B) Representative images of coronal sections of hippocampus showing dCA3 labeled with CaMKII‐ChR2 expression (red) and stained with DAPI (blue). Low magnification image, scale bar: 1 mm; High magnification image, scale bar: 100 μm. (C) Representative coronal immunofluorescent section showing the medial septum (MS) and the vertical diagonal band (VDB) projecting from dCA3 CaMKII^+^ neurons. Scale bar: 1 mm. (D) Enlarged images of the MS and VDB highlighting the details. Scale bar: 100 μm. (E) Representative coronal immunofluorescent section showing the dLS (dorsal lateral spetum) projecting from dCA3 CaMKII^+^ neurons. Scale bar: 1 mm. (F) Enlarged images of dLS highlighting the details. Scale bar: 100 μm. (G) Representative coronal immunofluorescent section showing the dCA1 (dorsal CA1) projecting from dCA3 CaMKII+ neurons. Scale bar: 1 mm. (H) Enlarged images of dCA1 highlighting the details. Scale bar: 100 μm. (I) Representative coronal immunofluorescent section showing the vCA3 (ventral CA3) projecting from dCA3. Scale bar: 1 mm. (J) Enlarged images of vCA3 highlighting the details. Scale bar: 100 μm. *n* = 3 mice subjected to the same manipulation exhibited similar morphological results.

We next aimed to understand how dCA3 CaMKII^+^ pyramidal neurons regulate cognitive flexibility by mapping the downstream connections of the dorsal CA3 (dCA3) region. To achieve this, we injected AAV2/9‐CaMKII‐ChR2‐mCherry‐WPRE‐pA into the dCA3 on one side of the hippocampus for anterograde tracing (Figure 6A,B). Our observations revealed that dCA3 primarily projects to ipsilateral hippocampal regions, including dCA1, dCA2, and vCA3. Interestingly, contralateral dCA1 also receives inputs from ipsilateral dCA3 neuronal axons (Figure 6G,I). Previous studies have demonstrated distinct neural activity in the left and right hippocampi during working memory tasks [18], suggesting that dCA3 (ipsilateral)‐dCA1 (contralateral) and dCA3 (ipsilateral)‐dCA1 (ipsilateral) circuits may serve different cognitive functions. Whether the contralateral dCA1 afferents originate from the same dCA3 neurons projecting to ipsilateral dCA1 requires further investigation. Additionally, the dCA3‐dCA1 circuit is involved in mediating the discrimination between threatening and safe contexts [19], which may facilitate the differentiation between new and familiar environments and enhance cognitive flexibility. Notably, dCA3 also projects directly to specific basal forebrain areas, including the dorsolateral septum (dLS), medial septum (MS), and vertical diagonal band (VDB) (Figure 6C–J). Given that these regions also send reciprocal projections back to dCA3 [20], it is plausible that the hippocampus forms a feedback network that synergistically modulates cognitive flexibility.

## 4 Discussion

In this study, we demonstrated that prolonged systemic exposure to IL‐1β significantly impairs cognitive flexibility in the BMT, with a notable portion of subjects showing resilience to this inflammation‐induced cognitive deficit during the reversal test phase of the BMT. Our findings indicate that the dCA3 region of the hippocampus is specifically involved in mediating this cognitive impairment. Activation of dCA3 neurons can reverse the cognitive inflexibility caused by inflammation, supporting the hypothesis that dCA3 pyramidal neurons are crucial for cognitive adaptation to changing spatial environments, particularly under inflammatory conditions. Furthermore, peripheral administration of IL‐1β did not affect general motor activity or anxiety‐like behavior in young adult C57BL/6J mice (3–4 months old). Additionally, cognitive processes such as memory acquisition, consolidation, and retrieval were unaffected, highlighting the functional heterogeneity of distinct hippocampal subregions in contributing to various hippocampus‐dependent memory processes.

The effects of IL‐1β on learning and memory are variable. For instance, one study found that small interfering RNA‐mediated IL‐1β knockdown in dorsal CA1 significantly prevented the aberrant recognition index induced by LPS administration in a novel object task [21]. In contrast, another study reported that IL‐1β administration reduced freezing time in contextual fear memory but did not affect cued fear memory [15]. In our study, we found that IL‐1β administration (1 μg/kg, *i.p*.) during an aversion‐associated spatial learning task (BMT) did not impair learning and memory but significantly attenuated cognitive flexibility in susceptible animals. This protective effect on spatial memory formation may be attributed to the possibility that prolonged cognitive training (repeat learning) enhances memory capacity against systemic inflammation [22]. Additionally, previous research has shown that aseptic inflammation induced by surgery and anesthesia does not affect the learning process or memory retrieval in young adult mice [23]. Similarly, a single intraperitoneal administration of IL‐1β (100 ng/kg or 1 μg/kg) does not disrupt these memory processes [24, 25]. Our findings suggest that even under inflammation‐induced distress, resilient individuals can develop adaptive coping strategies to overcome cognitive inflexibility. Future studies aimed at understanding the synaptic and molecular mechanisms involved should provide novel insights into how individuals adapt their behavioral responses to internal and external stressful challenges.

Cognitive flexibility is the ability to adapt an organism's behavior to changing environments, reflecting an easier switch from a situational goal to a novel scenario [26, 27]. Nevertheless, not all coping strategies are effective against stress, as different situations require tailored approaches. This underscores the importance of flexibility in stress responses, where adapting strategies to specific contexts is vital for resilience [28]. Previous research has identified the prefrontal cortex, posterior parietal cortex, and hippocampus as key regions involved in regulating cognitive flexibility [29, 30]. However, the specific hippocampal regions implicated in inflammation‐induced cognitive inflexibility remain unclear. The hippocampus comprises various subfields, including CA1, CA2, CA3, and the DG, each responsible for distinct cognitive functions (RE). Hippocampal neurogenesis in the dentate gyrus (DG) has been suggested to regulate cognitive flexibility by allowing adult‐born neurons to inhibit mature granule cells [31, 32, 33]. This process facilitates the formation of new memories associated with changing environments while erasing previously established context‐related memories. Intriguingly, our immunostaining results indicated that DG neuronal activity remained unchanged between resilient and susceptible subjects during reversal learning in the context of inflammation, consistent with the finding that chemogenetic silencing of CaMKII^+^ neurons within the DG delayed reaching the escape hole without affecting memory retrieval or cognitive flexibility. One plausible explanation is that the perforant path or external inputs to the hippocampus sustain the functional integrity of the CA1 and CA3 subfields, thereby exerting compensatory roles. Indeed, with DG lesions, hippocampus‐dependent mnemonic functions remain largely unaltered [17]. Additionally, chemogenetic silencing of DG did not alter the cognitive flexibility performance of the animals, which appears to contradict previous studies indicating that hyperexcitation of the DG caused by the deletion of adult‐born neurons leads to cognitive inflexibility [32, 33]. However, since the DG typically employs a sparse coding strategy for pattern separation—facilitating cognitive flexibility through neurogenesis—global silencing of DG pyramidal neurons may further impair the DG's ability to differentiate between similar conflicting conditions, thereby exacerbating cognitive inflexibility.

Evidence indicates that the hippocampal CA3 region, which receives direct input from the DG, plays a significant role in spatial tuning, remapping, and cognitive flexibility encoding and is involved in the rapid formation of a conjunctive and associative representation of a novel environment [5, 34]. Optogenetic activation of CA3 pyramidal neurons has been shown to enhance short‐term memory in APPS/PS1 mice [35]. Nakazawa et al. [36] found that the ablation of NMDA receptors in CA3 pyramidal neurons impaired the rapid acquisition of changing spatial information. Additionally, Ventura's work employed an environmental enrichment procedure to elucidate CA3's facilitative role in cognitive flexibility, revealing that increased activation of CA3 pyramidal neurons enhances spatial tuning related to novel information [37]. This underscores the involvement of CA3 pyramidal neurons in encoding rapidly changing environments and suggests a significant contribution to the regulation of cognitive flexibility. Our results indicated that inhibition of dCA3 pyramidal neurons significantly reduces motivation for spatial exploration in an updated environmental context. Furthermore, activation of dCA3 pyramidal neurons enhances exploratory behavior in the OFT without inducing anxiety‐like responses, aligning with the observed increase in calcium signal intensity when animals approach the target zone during the reversal stage compared to the retrieval stage. We propose that inflammation impairs neurogenesis in the DG, disrupting its functional role in facilitating reversal learning in both resilient and susceptible animals, which manifests as a “floor effect”. In contrast, CA3 neuronal activation was initiated as a compensatory mechanism in resilient individuals to maintain flexible execution of the reversal learning process.

While our study revealed an effect of dCA3 activation on alleviating reversal learning impairment induced by systemic inflammation, the initial formation and consolidation processes of the spatial memory were not impaired when dCA3 was inhibited. Thus, we could not support the hypothesis that dCA3 is recruited when forming a new representation under inflammation or that the effect was undetectable by current manipulation but instead seems to be specifically recruited in cognitively resilient individuals when representations need to be updated to adapt to the changing environments. Recent work by Dupret's lab indicated that optogenetic suppression of CA3 pyramidal neurons can restore flexible object‐location memory disrupted by previously formed contextual associations [38]. This discrepancy suggests that the role of CA3 in regulating cognitive flexibility is context‐sensitive and pattern‐specific. Further investigation is needed to determine whether the differential functional roles of CA3 pyramidal neurons are mediated by subtype‐specific or circuit‐specific neuronal populations and to identify molecular markers within the dCA3 region related to cognitive flexibility.

Hippocampal subregions differently regulate cognitive flexibility, depending on the orchestration of dynamic brain circuits and neural network patterns [34, 39]. Under the circumstance of systemic inflammation, alterations in the functional and anatomical circuits also occur [40, 41, 42]. Studies have displayed that surgery‐ or anesthesia‐induced inflammation significantly inhibits the IL‐BLA pathway while activating the IL‐BMA circuit. Manipulation of the medial prefrontal cortex‐amygdala glutamatergic pathway has been linked to postoperative cognitive dysfunction [43]. Additionally, both the ventral tegmental area (VTA) and lateral habenula (LHb) are activated postoperatively, and silencing the LHb‐VTA circuit has been shown to alleviate cognitive impairments and reverse reduced dendritic spine density in the prefrontal cortex and hippocampus [44]. These findings suggest that inflammation‐induced cognitive deficits resulting from stressors, including surgery, anesthesia, and cytokines, involve both intra‐ and extra‐hippocampal regions. Notably, our investigation revealed that the dorsal CA3 region projects to several brain areas implicated in cognitive functions, including the dorsal lateral septum and dCA1. Previous studies have reported that dCA3 receives synaptic inputs from the DG, locus coeruleus, and other regions [45, 46], indicating that dCA3 is a critical node in regulating cognitive flexibility. It is plausible that other components of the limbic system also contribute to the regulation of cognitive flexibility under inflammatory conditions. Further investigation is needed to identify the neural networks, particularly the distinct connections of CA3 and other relevant brain areas that are functionally involved in mediating various cognitive processes during inflammation [47].

## 5 Conclusions

In conclusion, our findings demonstrate that systemic IL‐1β administration influences exploratory strategies and cognitive flexibility in vulnerable animals. Specifically, activated dCA3 pyramidal neurons play a crucial role in promoting cognitive resilience during the reversal stage of the BMT in the context of inflammation. These results support the notion that dorsal CA3 pyramidal neuronal activity is significantly involved in regulating the behavioral response to systemic inflammation‐induced stress. The activation of these neurons can mitigate cognitive inflexibility associated with systemic inflammation. Our study presents a promising avenue for enhancing resilience against inflammation‐related cognitive deficits and suggests a potential therapeutic target for ameliorating mental illnesses characterized by cognitive inflexibility.

## Author Contributions

M.W., W.H., and W.Z. contributed to the conceptualization; M.W. and W.H. supervised the project; M.W. and W.Z. wrote the manuscript; W.Z., S.F., T.X., Y.L., J.Z., X.X., and T.W. performed the experiments; W.Z., S.F., and T.X. analyzed the data. All authors reviewed the manuscript.

## Conflicts of Interest

The authors declare no conflicts of interest.

## Supporting information


**Figure S1.** Consecutive exposure to IL‐1β did not influence general locomotion or blood–brain barrier integrity. (A) Schematic representation of the experimental timeline. (B) Track plots from the open field test for vehicle‐ and IL‐1β‐treated male mice on days 1 and 5. (C) Quantification of total distance traveled in open field test for vehicle‐ or IL‐1β‐treated male mice on days 1 and 5 (rmTwo‐way ANOVA, Group: *F*_(1,14)_ = 0.9138, *p* = 0.3553) (left panel), and quantification of time spent in the center zone (light blue) for vehicle‐ or IL‐1β‐treated male mice on days 1 and 5 (rmTwo‐way ANOVA, Group: *F*_(1,14)_ = 0.1819, *p* = 0.6762) (right panel). (D) Track plots from the open field test for vehicle‐ and IL‐1β‐treated female mice on days 1 and 5. (E) Quantification of total distance traveled in open field test for vehicle‐ or IL‐1β‐treated female mice on days 1 and 5 (rmTwo‐way ANOVA, Group: *F*_(1,14)_ = 4.173, *p* = 0.0604) (left panel), and quantification of time spent in the center zone (light blue) for vehicle‐ or IL‐1β‐treated female mice on days 1 and 5 (rmTwo‐way ANOVA, Group: *F*_(1,14)_ = 0.04020, *p* = 0.8440) (right panel). *n* = 8 mice per group. (F) The integrity of the blood–brain barrier was evaluated using Evans blue staining in vehicle‐ or IL‐1β‐treated mice. *n* = 4 mice per group.

**Figure S2.** Systemic exposure to IL‐1β did not impair cognitive flexibility in female mice. (A) Schematic representation of the experimental timeline. (B) Latency to explore the escape hole during memory acquisition for female mice (rm Two‐way ANOVA, Group: *F*_(1,18)_ = 0.1397, *p* = 0.7129). (C) Average speed during memory acquisition (rm Two‐way ANOVA, Group: *F*_(1,18)_ = 0.8274, *p* = 0.3751). (D) Behavioral track plots during memory acquisition for vehicle‐ and IL‐1β‐treated female mice. (E) Analysis of different searching strategies (left panel) and searching strategy (non‐random or random) in mice exposed to IL‐1β or vehicle during acquisition (right panel) (Fisher’s exact test, *p* = 0.1450). (F) Time spent (%) in different quadrants during the probe trial. (Two‐way ANOVA, Group: *F*_(1,72)_ = 0.0003307, *p* = 0.9855). (G) Sample track heatmaps of vehicle‐ and IL‐1β‐treated female mice during the probe trial. (H) Latency to reach the reversal hole was measured in vehicle‐ and IL‐1β‐treated female mice during the BMT. The red dashed line indicates the cut‐off value, calculated as the mean plus one standard deviation (SD) (unpaired Student’s *t*‐test, *p* = 0.1336). (I) Time(s) spent in the target zone of vehicle‐ and IL‐1β‐treated female mice during the reversal stage of BMT (Mann–Whitney test, *p* = 0.1163). (J) Track plots of the vehicle or IL‐1β groups during the reversal stage (left panel), along with the proportion of resilient and susceptible mice (right panel) (Fisher’s exact test, *p* = 0.6424). *n* = 11 mice for the vehicle group, *n* = 9 mice for the IL‐1β group. All values are presented as mean ± SEM.

**Figure S3.** Demonstration of c‐Fos expression profiling in the dorsal and ventral hippocampal subfields of cognitively resilient and susceptible mice. (A) Schematic illustration showing coronal brain sections along with representative images of c‐Fos expression in the dorsal hippocampus of naive, resilient, and susceptible mice, spanning from 1.34 to 2.80 mm posterior to bregma. DAPI is shown in blue, and c‐Fos is shown in green. Scale bar: 200 μm. (B) Schematic illustration showing coronal brain sections along with representative images of c‐Fos expression in the ventral hippocampus of naive, resilient, and susceptible mice, spanning from −3.08 to −3.52 mm posterior to bregma. DAPI is shown in blue, c‐Fos is shown in green Scale bar: 500 μm.

**Figure S4.** Colocalization of inflammation‐induced c‐Fos and CaMKII expression in the DG. (A) Representative images displaying immunostaining for CaMKII (red), GABA (cyan), c‐Fos (green), and DAPI (blue). (B) Quantification of colocalization between c‐Fos^+^ and CaMKII^+^ cells in the DG of naive, resilient, and susceptible mice (one‐way ANOVA, *F*_(2,6)_ = 1.650, *p* = 0.2685). All values are presented as mean ± SEM.

**Figure S5.** Effect of chemogenetic modulation of dCA3 CaMKII^+^ neurons on locomotion in the open field test. (A) Track plots from the open field test for mCh + Sal, mCh + CNO, 3Dq + Sal, and 3Dq + CNO mice. (B) Quantification of total distance traveled (one‐way ANOVA, *F*_(3,27)_ = 4.488, *p* = 0.0111) (left) and time spent in the center zone (right) in the open field test for mice with mCherry or hM4Di expressed in the dCA3 and treated with Saline or CNO (Kruskal–Wallis test, *H* = 0.8675). *n* = 8 mice for mCh + Sal; *n* = 8 mice for mCh + CNO; *n* = 7 mice for 3Dq + Sal; *n* = 8 mice for 3Dq + CNO. (C) Track plots from the open field test across different experimental groups. (D) Quantification of total distance traveled (One‐way ANOVA, *F*_(3,28)_ = 0.8880, *p* = 0.4594) (left) and time spent in the center zone (right) in open field test for mice with hM4Di in the dCA3 or DG and treated with CNO (One‐way ANOVA, *F*_(3,28)_ = 0.6960, *p* = 0.5623). *n* = 8 mice per group. (E) The representative image of the ex vivo recording and the corresponding electrophysiological trace demonstrate that the application of CNO (1 μM) on hM4Di‐infected dCA3 neurons inhibits neuronal spiking. (F) Representative image of ex vivo recording and the corresponding electrophysiological trace demonstrate that the application of CNO (1 μM) on hM4Di‐infected DG neurons inhibits neuronal spiking. Created with Biorender.com. *p < 0.05. All values are presented as mean ± SEM.

**Figure S6.** Chemogenetic inhibition of CaMKII^+^ neurons in the DG did not impair cognitive flexibility. (A) Schematic representation of bilateral viral injection in the DG. Created with Biorender.com. (B) Representative images of CaMKII‐Cre (green) dependent hM4Di expression (red) in DG and stained with DAPI (blue). (C) Schematic diagram showing the experiment timeline of the Barnes maze test for mCherry‐ or hM4Di‐injected mice treated with CNO. (D) Latency to explore the escape hole during the memory acquisition phase of BMT (rm Two‐way ANOVA, Group: *F*_(1,16)_ = 5.558, *p* = 0.0315). (E) Average speed to explore the platform during the memory acquisition phase of BMT (rm Two‐way ANOVA, Group: *F*_(1,16)_ = 1.996, *p* = 0.1769). (F) Track plots of BMT behavioral test for mCherry‐ or hM4Di‐injected mice treated with CNO during the memory acquisition. (G) Proportions of different searching strategies during the memory acquisition phase of BMT. (H) Comparison of non‐random and random searching strategies during acquisition phase of BMT for mCherry‐ or hM4Di‐injected mice treated with CNO (Fisher’s exact test, *p* = 0.0179). (I) Percentage of time spent in different quadrants in probe trial for mCherry‐ or hM4Di‐injected mice treated with CNO (Two‐way ANOVA, treatment: *F*_(1,64)_ = 0.02749, *p* = 0.8688), (J) Heatmaps of representative mice in the probe trial for mCherry‐ or hM4Di‐injected mice treated with CNO. (K) Latency to reach the reversal hole during the reversal stage of BMT. The red dashed line indicates the cut‐off value, calculated as the mean plus one SD (Mann–Whitney test, *p* = 0.4894). (L) Time in the target zone for mCherry‐ or hM4Di‐injected mice treated with CNO during the reversal stage of BMT (Mann–Whitney test, *p* = 0.3284). (M) Track plots (left) of mCherry‐ or hM4Di‐injected mice treated with CNO during the reversal stage of BMT, and the proportion of resilient and susceptible mice (right) (Fisher’s exact test, *p* > 0.9999). *p < 0.05; **p < 0.01; ****p < 0.0001. *n* = 9 mice per group. Scale bar: 200 μm. All values are presented as mean ± SEM.

**Table S1.** All statistical information for Figure 1 to Figure S6.

## Data Availability

The data used or analyzed during the current study are available from the corresponding author upon reasonable request.
