# Supplementary figures and images for: CA3 Pyramidal Neuron Activation Promotes Cognitive Resilience to Inflammation‐Induced Cognitive Inflexibility
Source: CNS Neurosci Ther. 2025 Feb 25;31(2):e70271. doi: 10.1111/cns.70271 (PMC11851139; doi:10.1111/cns.70271)

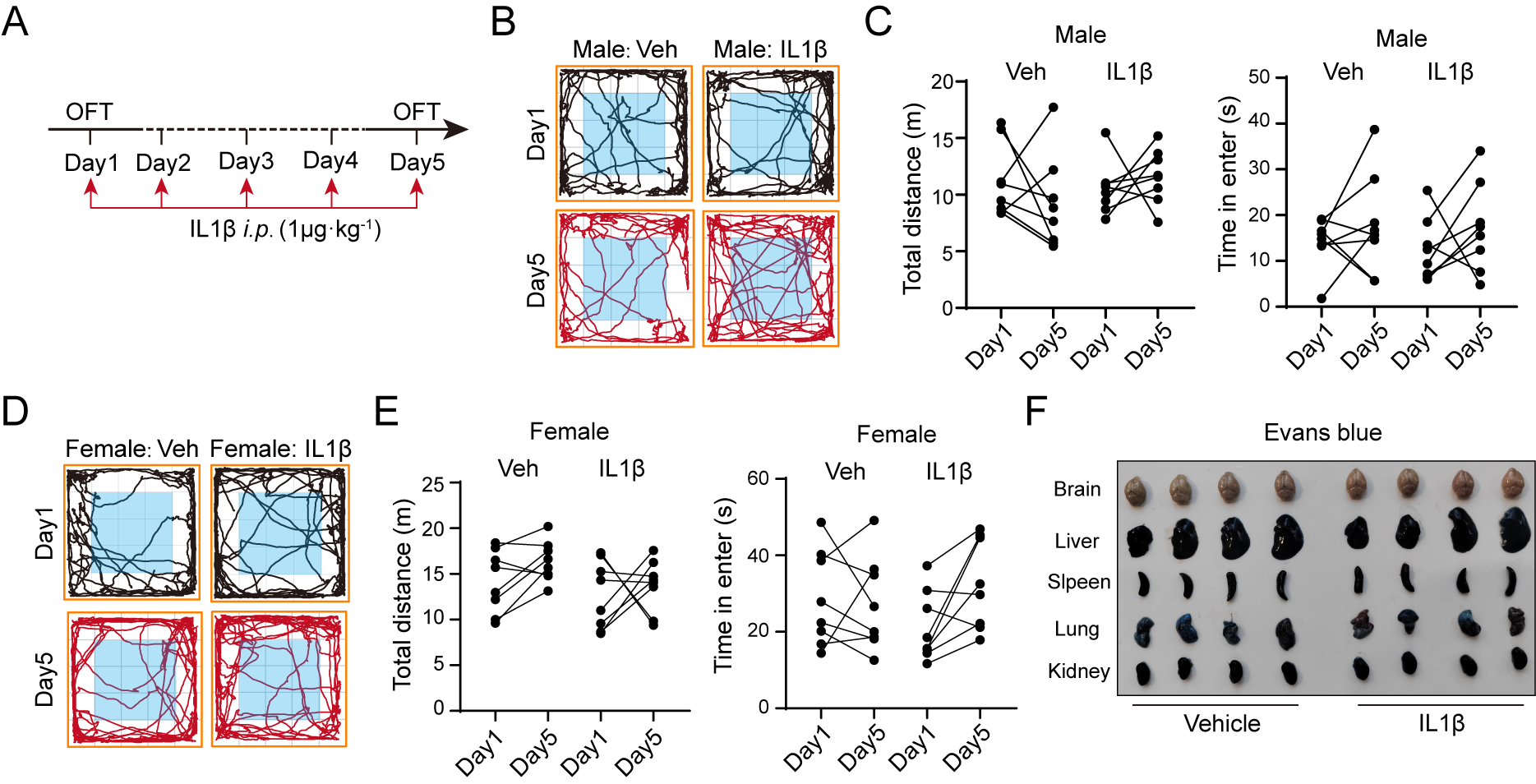

Supplement: Supplementary file 1 — Figure S1. Consecutive exposure to IL‐1β did not influence general locomotion or blood–brain barrier integrity. (A) Schematic representation of the experimental timeline. (B) Track plots from the open field test for vehicle‐ and IL‐1β‐treated male mice on days 1 and 5. (C) Quantification of total distance traveled in open field test for vehicle‐ or IL‐1β‐treated male mice on days 1 and 5 (rmTwo‐way ANOVA, Group: F (1,14) = 0.9138, p = 0.3553) (left panel), and quantification of time spent in the center zone (light blue) for vehicle‐ or IL‐1β‐treated male mice on days 1 and 5 (rmTwo‐way ANOVA, Group: F (1,14) = 0.1819, p = 0.6762) (right panel). (D) Track plots from the open field test for vehicle‐ and IL‐1β‐treated female mice on days 1 and 5. (E) Quantification of total distance traveled in open field test for vehicle‐ or IL‐1β‐treated female mice on days 1 and 5 (rmTwo‐way ANOVA, Group: F (1,14) = 4.173, p = 0.0604) (left panel), and quantification of time spent in the center zone (light blue) for vehicle‐ or IL‐1β‐treated female mice on days 1 and 5 (rmTwo‐way ANOVA, Group: F (1,14) = 0.04020, p = 0.8440) (right panel). n = 8 mice per group. (F) The integrity of the blood–brain barrier was evaluated using Evans blue staining in vehicle‐ or IL‐1β‐treated mice. n = 4 mice per group. [file CNS-31-e70271-s003.tif]

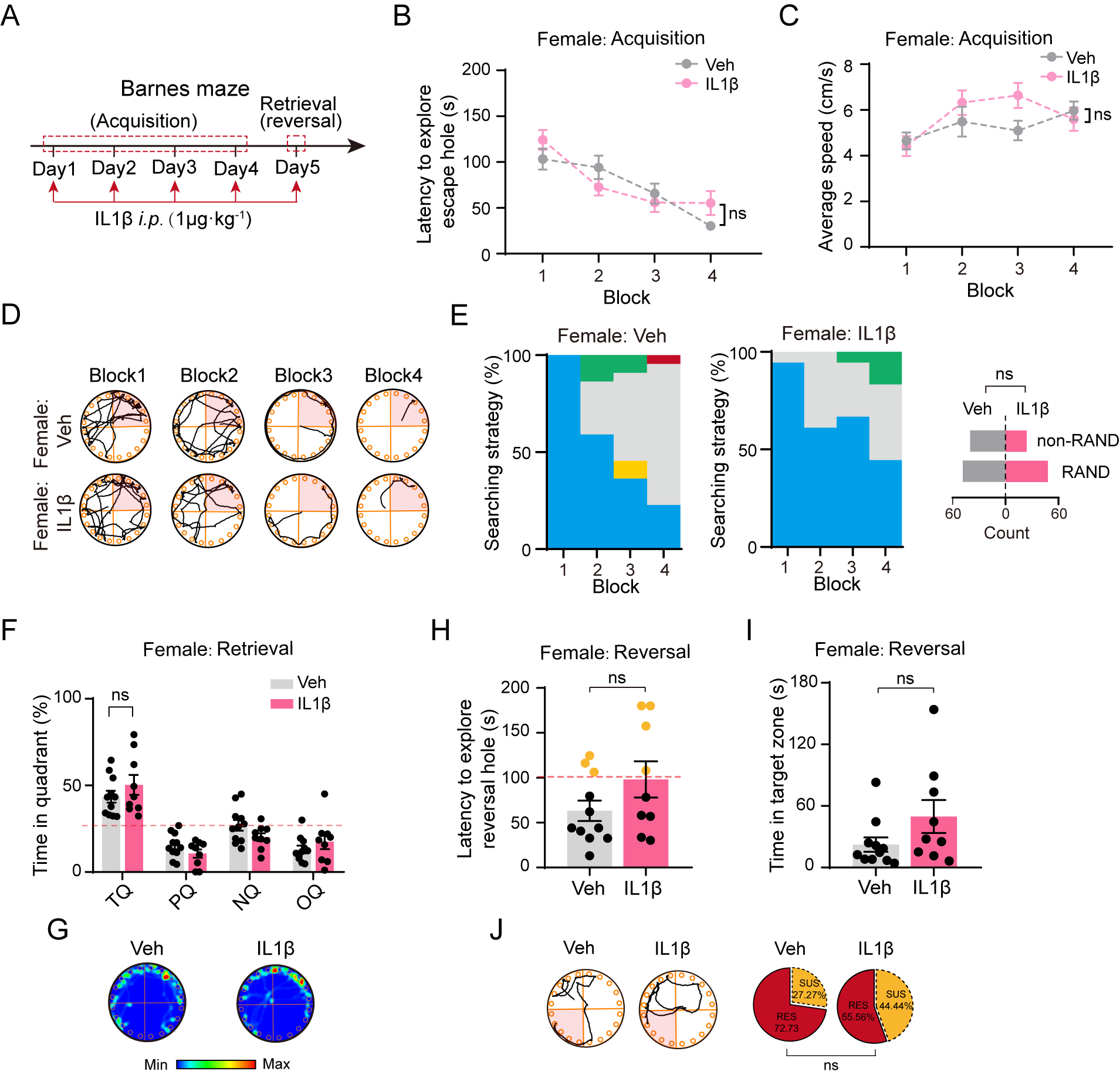

Supplement: Supplementary file 2 — Figure S2. Systemic exposure to IL‐1β did not impair cognitive flexibility in female mice. (A) Schematic representation of the experimental timeline. (B) Latency to explore the escape hole during memory acquisition for female mice (rm Two‐way ANOVA, Group: F (1,18) = 0.1397, p = 0.7129). (C) Average speed during memory acquisition (rm Two‐way ANOVA, Group: F (1,18) = 0.8274, p = 0.3751). (D) Behavioral track plots during memory acquisition for vehicle‐ and IL‐1β‐treated female mice. (E) Analysis of different searching strategies (left panel) and searching strategy (non‐random or random) in mice exposed to IL‐1β or vehicle during acquisition (right panel) (Fisher’s exact test, p = 0.1450). (F) Time spent (%) in different quadrants during the probe trial. (Two‐way ANOVA, Group: F (1,72) = 0.0003307, p = 0.9855). (G) Sample track heatmaps of vehicle‐ and IL‐1β‐treated female mice during the probe trial. (H) Latency to reach the reversal hole was measured in vehicle‐ and IL‐1β‐treated female mice during the BMT. The red dashed line indicates the cut‐off value, calculated as the mean plus one standard deviation (SD) (unpaired Student’s t‐test, p = 0.1336). (I) Time(s) spent in the target zone of vehicle‐ and IL‐1β‐treated female mice during the reversal stage of BMT (Mann–Whitney test, p = 0.1163). (J) Track plots of the vehicle or IL‐1β groups during the reversal stage (left panel), along with the proportion of resilient and susceptible mice (right panel) (Fisher’s exact test, p = 0.6424). n = 11 mice for the vehicle group, n = 9 mice for the IL‐1β group. All values are presented as mean ± SEM. [file CNS-31-e70271-s001.tif]

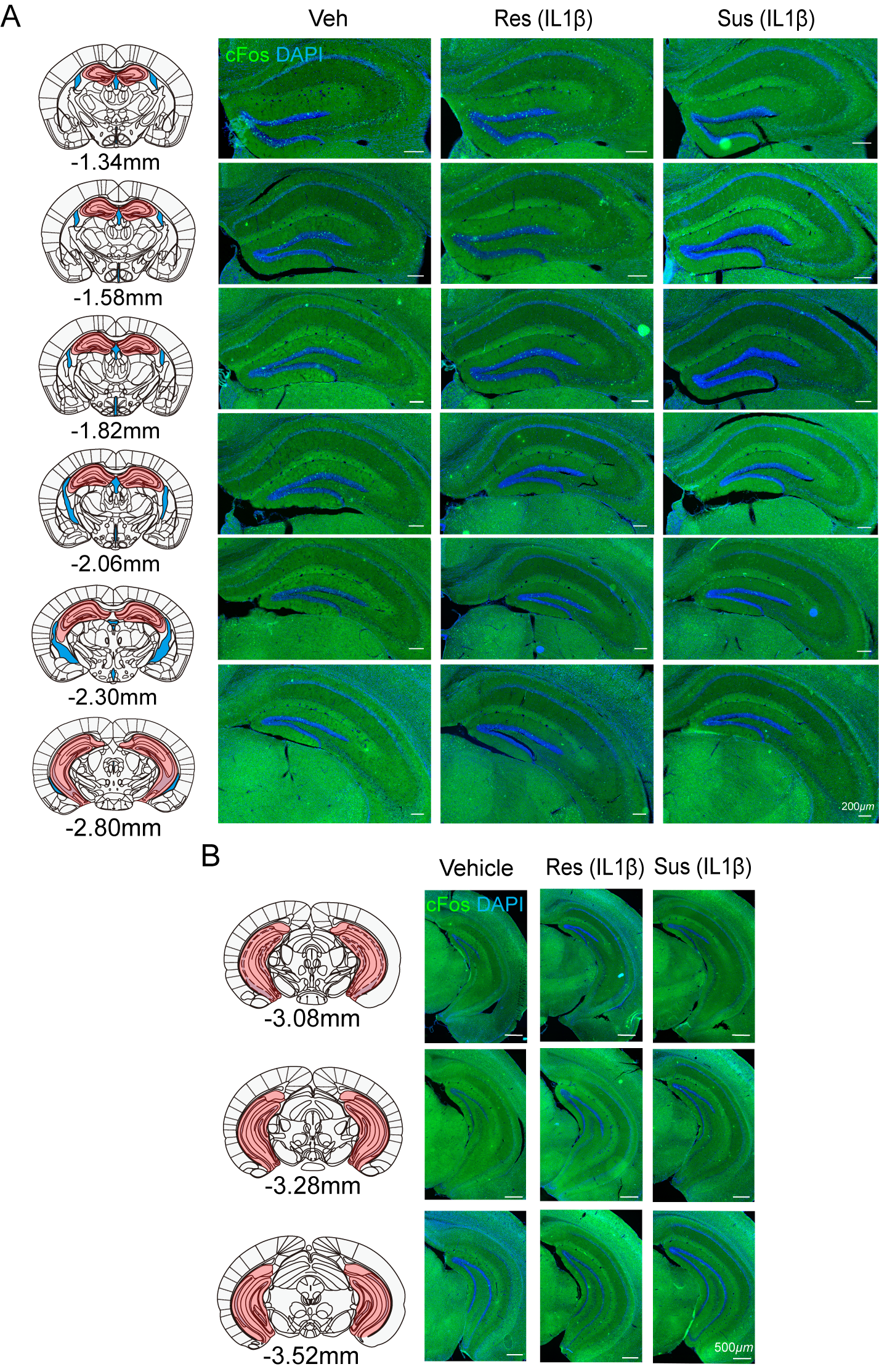

Supplement: Supplementary file 3 — Figure S3. Demonstration of c‐Fos expression profiling in the dorsal and ventral hippocampal subfields of cognitively resilient and susceptible mice. (A) Schematic illustration showing coronal brain sections along with representative images of c‐Fos expression in the dorsal hippocampus of naive, resilient, and susceptible mice, spanning from 1.34 to 2.80 mm posterior to bregma. DAPI is shown in blue, and c‐Fos is shown in green. Scale bar: 200 μm. (B) Schematic illustration showing coronal brain sections along with representative images of c‐Fos expression in the ventral hippocampus of naive, resilient, and susceptible mice, spanning from −3.08 to −3.52 mm posterior to bregma. DAPI is shown in blue, c‐Fos is shown in green Scale bar: 500 μm. [file CNS-31-e70271-s005.tif]

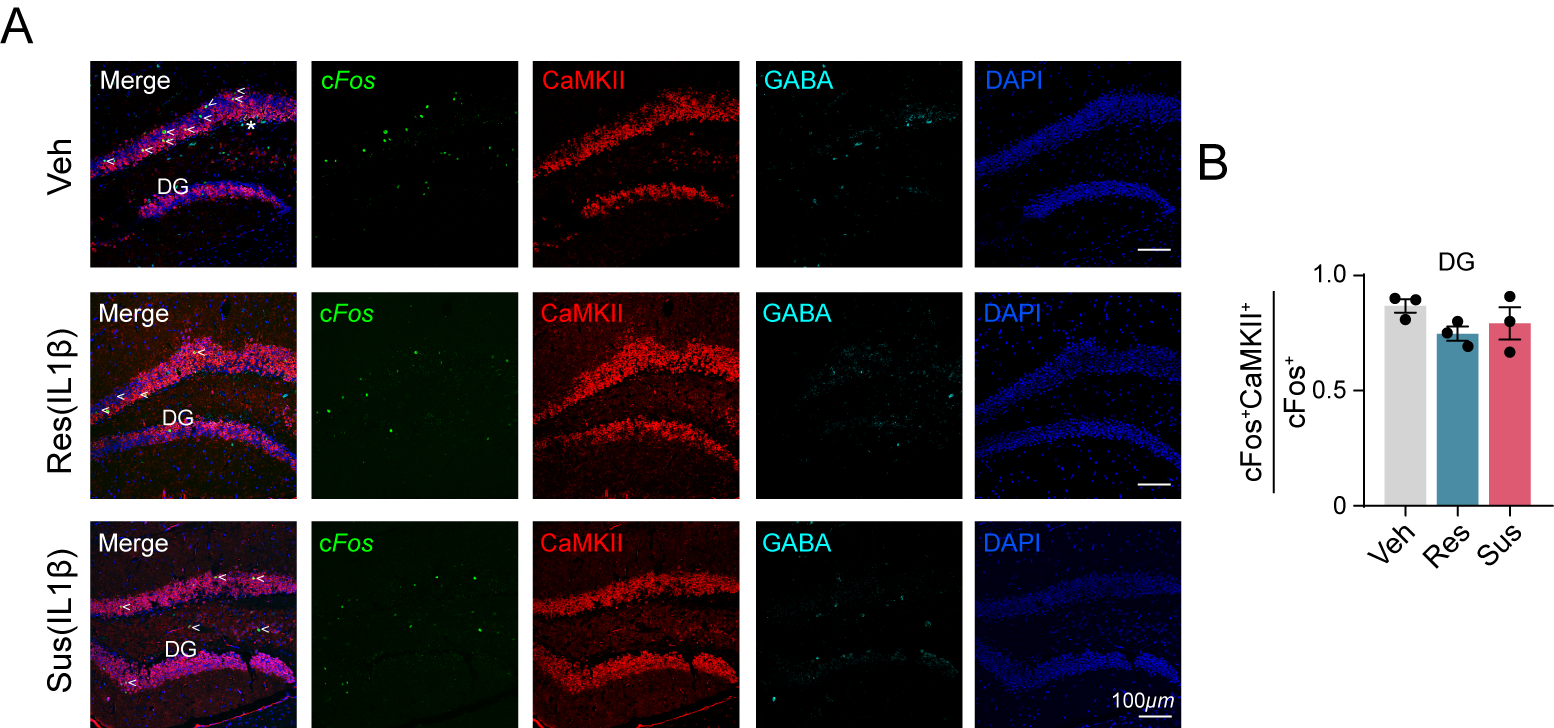

Supplement: Supplementary file 4 — Figure S4. Colocalization of inflammation‐induced c‐Fos and CaMKII expression in the DG. (A) Representative images displaying immunostaining for CaMKII (red), GABA (cyan), c‐Fos (green), and DAPI (blue). (B) Quantification of colocalization between c‐Fos+ and CaMKII+ cells in the DG of naive, resilient, and susceptible mice (one‐way ANOVA, F (2,6) = 1.650, p = 0.2685). All values are presented as mean ± SEM. [file CNS-31-e70271-s004.tif]

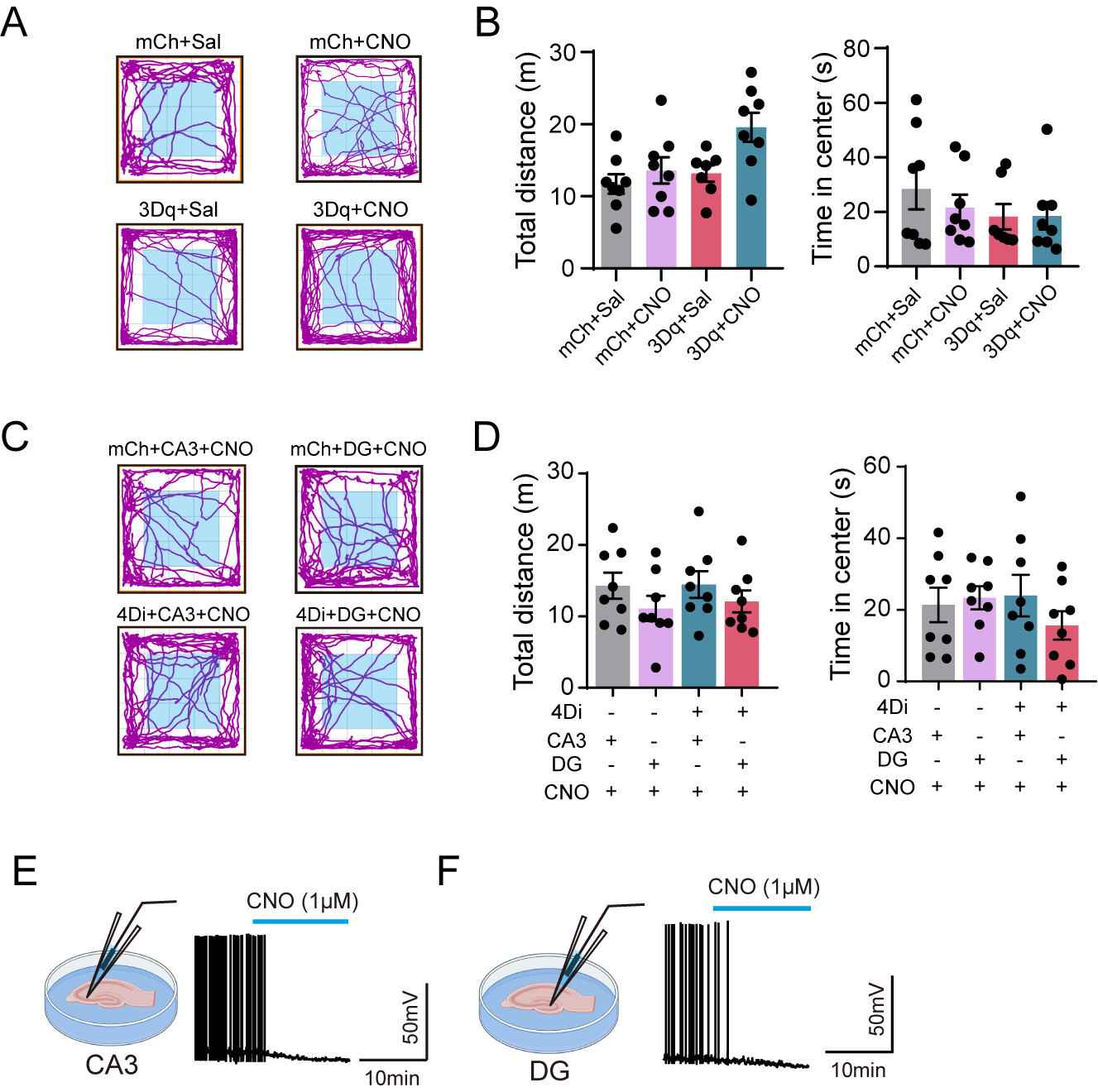

Supplement: Supplementary file 5 — Figure S5. Effect of chemogenetic modulation of dCA3 CaMKII+ neurons on locomotion in the open field test. (A) Track plots from the open field test for mCh + Sal, mCh + CNO, 3Dq + Sal, and 3Dq + CNO mice. (B) Quantification of total distance traveled (one‐way ANOVA, F (3,27) = 4.488, p = 0.0111) (left) and time spent in the center zone (right) in the open field test for mice with mCherry or hM4Di expressed in the dCA3 and treated with Saline or CNO (Kruskal–Wallis test, H = 0.8675). n = 8 mice for mCh + Sal; n = 8 mice for mCh + CNO; n = 7 mice for 3Dq + Sal; n = 8 mice for 3Dq + CNO. (C) Track plots from the open field test across different experimental groups. (D) Quantification of total distance traveled (One‐way ANOVA, F (3,28) = 0.8880, p = 0.4594) (left) and time spent in the center zone (right) in open field test for mice with hM4Di in the dCA3 or DG and treated with CNO (One‐way ANOVA, F (3,28) = 0.6960, p = 0.5623). n = 8 mice per group. (E) The representative image of the ex vivo recording and the corresponding electrophysiological trace demonstrate that the application of CNO (1 μM) on hM4Di‐infected dCA3 neurons inhibits neuronal spiking. (F) Representative image of ex vivo recording and the corresponding electrophysiological trace demonstrate that the application of CNO (1 μM) on hM4Di‐infected DG neurons inhibits neuronal spiking. Created with Biorender.com. *p < 0.05. All values are presented as mean ± SEM. [file CNS-31-e70271-s002.tif]

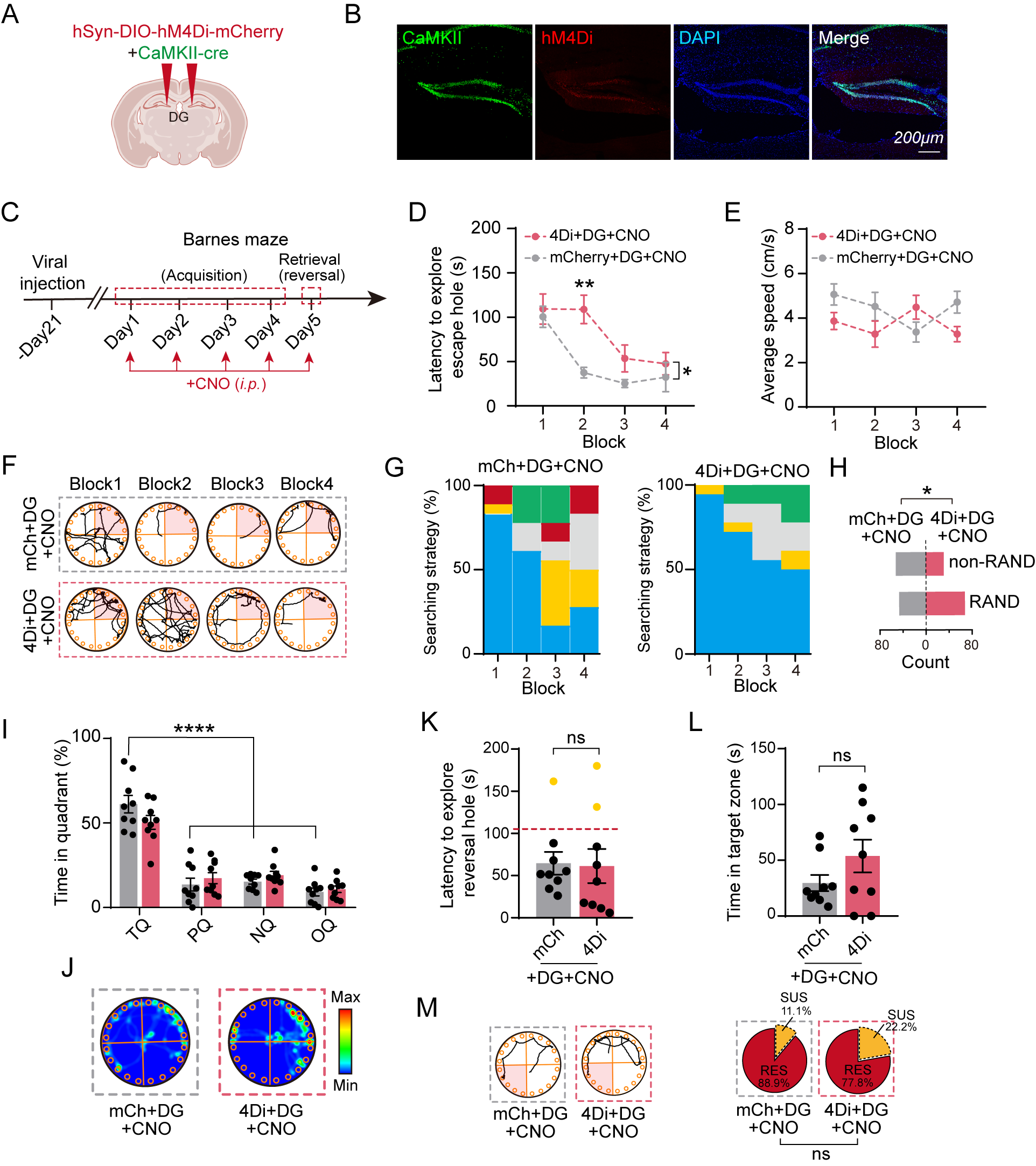

Supplement: Supplementary file 6 — Figure S6. Chemogenetic inhibition of CaMKII+ neurons in the DG did not impair cognitive flexibility. (A) Schematic representation of bilateral viral injection in the DG. Created with Biorender.com. (B) Representative images of CaMKII‐Cre (green) dependent hM4Di expression (red) in DG and stained with DAPI (blue). (C) Schematic diagram showing the experiment timeline of the Barnes maze test for mCherry‐ or hM4Di‐injected mice treated with CNO. (D) Latency to explore the escape hole during the memory acquisition phase of BMT (rm Two‐way ANOVA, Group: F (1,16) = 5.558, p = 0.0315). (E) Average speed to explore the platform during the memory acquisition phase of BMT (rm Two‐way ANOVA, Group: F (1,16) = 1.996, p = 0.1769). (F) Track plots of BMT behavioral test for mCherry‐ or hM4Di‐injected mice treated with CNO during the memory acquisition. (G) Proportions of different searching strategies during the memory acquisition phase of BMT. (H) Comparison of non‐random and random searching strategies during acquisition phase of BMT for mCherry‐ or hM4Di‐injected mice treated with CNO (Fisher’s exact test, p = 0.0179). (I) Percentage of time spent in different quadrants in probe trial for mCherry‐ or hM4Di‐injected mice treated with CNO (Two‐way ANOVA, treatment: F (1,64) = 0.02749, p = 0.8688), (J) Heatmaps of representative mice in the probe trial for mCherry‐ or hM4Di‐injected mice treated with CNO. (K) Latency to reach the reversal hole during the reversal stage of BMT. The red dashed line indicates the cut‐off value, calculated as the mean plus one SD (Mann–Whitney test, p = 0.4894). (L) Time in the target zone for mCherry‐ or hM4Di‐injected mice treated with CNO during the reversal stage of BMT (Mann–Whitney test, p = 0.3284). (M) Track plots (left) of mCherry‐ or hM4Di‐injected mice treated with CNO during the reversal stage of BMT, and the proportion of resilient and susceptible mice (right) (Fisher’s exact test, p > 0.9999). *p < 0.05; **p < 0.01; ****p < 0.0001. n = [file CNS-31-e70271-s006.tif]
